# Determination of Selected Isoquinoline Alkaloids from *Mahonia aquifolia; Meconopsis cambrica; Corydalis lutea; Dicentra spectabilis; Fumaria officinalis; Macleaya cordata* Extracts by HPLC-DAD and Comparison of Their Cytotoxic Activity

**DOI:** 10.3390/toxins11100575

**Published:** 2019-10-02

**Authors:** Anna Petruczynik, Tomasz Plech, Tomasz Tuzimski, Justyna Misiurek, Barbara Kaproń, Dorota Misiurek, Małgorzata Szultka-Młyńska, Bogusław Buszewski, Monika Waksmundzka-Hajnos

**Affiliations:** 1 Department of Inorganic Chemistry, Medical University of Lublin, Chodźki 4a, 20-093 Lublin, Poland; justyna.misiurek@umlub.pl; 2 Department of Pharmacology, Medical University of Lublin, Chodźki 4a, 20-093 Lublin, Poland; tomasz.plech@umlub.pl; 3 Department of Physical Chemistry, Medical University of Lublin, Chodźki 4a, 20-093 Lublin, Poland; tomasz.tuzimski@umlub.pl; 4 Department of Clinical Genetics, Medical University of Lublin, Radziwiłłowska 11, 20-080 Lublin, Poland; barbara.kapron@umlub.pl; 5 Botanical Garden of Maria Curie-Skłodowska University in Lublin, Sławinkowska 3, 20-810 Lublin, Poland; dorota.misiurek@poczta.umcs.lublin.pl; 6 Department of Environmental Chemistry and Bioanalytics, Nicolaus Copernicus University, Faculty of Chemistry Gagarina 7, PL-87-100 Torun, Poland; szultka.malgorzata@wp.pl (M.S.-M.); bbusz@chem.umk.pl (B.B.)

**Keywords:** isoquinoline alkaloids, HPLC-DAD, cytotoxic activity, *Mahonia aquifolia*, *Meconopsis cambrica*, *Corydalis lutea*, *Dicentra spectabilis*, *Fumaria officinalis*, *Macleaya cordata*

## Abstract

Alkaloids have protective functions for plants and can play an important role in living organisms. Alkaloids may have a wide range of pharmacological activities. Many of them have cytotoxic activity. Nowadays, cancer has become a serious public health problem. Searching for effective drugs with anticancer activity is one of the most significant challenges of modern scientific research. The aim of this study was the investigation of cytotoxic activity of extracts obtained from *Corydalis lutea* root and herb, *Dicentra spectabilis* root and herb, *Fumaria officinalis*, *Macleaya cordata* leaves and herb, *Mahonia aquifolia* leaves and cortex, *Meconopsis cambrica* root and herb on FaDu, SCC-25, MCF-7, and MDA-MB-231 cancer cell lines. The cytotoxic activity of these extracts has not been previously tested for these cell lines. The aim was also to quantify selected alkaloids in the investigated extracts by High Performance Liquid Chromatography (HPLC). The analyses of alkaloid content were performed using HPLC in reversed phase (RP) mode using Polar RP column and mobile phase containing acetonitrile, water and ionic liquid (IL). Cytotoxic effect of the tested plant extracts and respective alkaloid standards were examined using human pharyngeal squamous carcinoma cells (FaDu), human tongue squamous carcinoma cells (SCC-25), human breast adenocarcinoma cell line (MCF-7), human triple-negative breast adenocarcinoma cell line (MDA-MB-231). All investigated plant extracts possess cytotoxic activity against tested cancer cell lines: FaDu, SCC-25, MCF-7, and MDA-MB-231. The highest cytotoxic activity against FaDu, SCC-25, and MCF-7 cell lines was estimated for *Macleaya cordata* leaf extract, while the highest cytotoxic activity against MDA-MB-231 cell line was obtained for *Macleaya cordata* herb extract. Differences in cytotoxic activity were observed for extracts obtained from various parts of investigated plants. In almost all cases the cytotoxic activity of investigated plant extracts, especially at the highest concentration against tested cell lines was significantly higher than the activity of anticancer drug etoposide. Our investigations exhibit that these plant extracts can be recommended for further in vivo experiments to confirm their anticancer activity.

## 1. Introduction

Cancer is one of the most prominent diseases in humans and currently there is considerable scientific interest shown towards the exploration of new anticancer agents from natural sources including plants. Many plants are the source of a variety of substances, including secondary metabolites which exhibit the anticancer activity. Most of the anticancer drugs obtained from plants inhibit the nucleic acid synthesis, but the mechanism of action differs widely.

Isoquinoline alkaloids are a group of natural bioactive products with widespread occurrence in nature. Some isoquinoline alkaloids have antibacterial, antifungal, anti-tumor and other biological activities. For the determination of them in plants, the modern chromatographic methods are most often applied.

Species of *Mahonia* are used in folk medicine worldwide as a cure for tuberculosis, dysentery, various skin disorders and showed antibacterial, antifungal, and anti-inflammatory properties [1]. The stem bark of *Mahonia aquifolium* contains a lot of isoquinoline alkaloids, including berberine, palmatine, jatrorrhizine, magnoflorine and berbamine [2]. Plant extracts obtained from various species of *Mahonia* genus were previously analyzed by HPLC most often on octadecyl stationary phases with mobile phases containing acetonitrile, water with the addition of formic acid [3], trifluoroacetic acid [4], sodium dihydrogen phosphate [5], phosphate buffer at pH 3.0 [6]. Previous investigations showed that representatives of genus *Mahonia*, such as *Mahonia balei*, *Mahonia oiwakensis* and *Mahonia aquifolium* have cytotoxic activity against various human cancer cells, e.g., human colon cancer (HT-29) [5], human lung adenocarcinoma cells (A549 and H1355) [1], large-cell lung carcinoma (H1299) [1], and squamous-cell carcinoma of the lung (H226) [1], human cervical adenocarcinoma cell line (HeLa) [2].

*Fumaria* species are sometimes used in herbal medicine. *Fumaria* extracts are components of several phytotherapeutic preparations, which are used mostly in cases of minor hepatobiliary dysfunction, gastrointestinal diseases, diuretic agents and for skin disorders [7]. Alkaloids in extracts obtained from various *Fumaria* species were determined usually on C18 columns. Mobile phases contained a mixture of acetonitrile, water with the addition of formic acid [7,8] was most often applied. Rarely, cytotoxic properties of extracts from *Fumaria* species were investigated. Anti-proliferative activity of *Fumaria vaillantii* extract was investigated on malignant melanoma SKMEL-3, human breast adenocarcinoma MCF-7 and human myelogenous leukemia K562 cells [9]. Anticancer activity of *Fumaria indica* was investigated on rat hepatic carcinoma cells [10]. Chemopreventive effect of the plant extract against N-nitrosodiethylamine and CCl_4_-induced hepatocellular carcinoma was determined.

*Macleaya cordata* has antiviral, anti-inflammatory, and insecticidal properties. The plant has been used to cure cervical cancer and thyroid cancer in traditional folk medicine [11]. The alkaloid analyses in extracts obtained from *Macleaya microcarpa, Macleaya cordata* were carried out most often by HPLC on C18 column using mobile phases containing acetonitrile, water, and formic acid [11,12,13,14]. Alkaloids in *Macleaya cordata* extract were also analyzed on (C12) column [15]. A mixture of acetonitrile, water, trimethylamine, and 1-heptanesulfonic acid was applied as mobile phase. Anticancer activity of *Macleaya* species was rarely investigated. Cytotoxic effects of *Macleaya cordata* extract was observed against adenocarcinomic lung cells [16]. The extract obtained from roots of *Macleaya microcarpa* exhibit cytotoxicity against Bel-7402, BGC-823, HCT-8, A2780, and A549 human cell-lines [17].

Several species of the genus Corydalis have been used over the past centuries in traditional Asian medicine. Corydalis sp. have antioxidant and anticancer activities, pain relief, and promotion of blood circulation pharmacological effects [18]. The major active constituents of Corydalis species are isoquinoline alkaloids: Berberine, palmatine, coptisine, isocorydine, and glaucine. These have a wide range of pharmacological activities such as: Antioxidant, antibacterial, antiviral, anticancer, analgesic, and promotion of blood circulation. For analysis of alkaloids in extracts obtained from *Corydalis* species, C18 column and mobile phases consisted of acetonitrile, water and formic acid [19,20,21,22], ammonium acetate [23], formic acid and ammonium acetate [24], acetate buffer at pH 5.0 [20], ammonium hydroxide [25], acetonitrile, water, acetic acid, and diethylamine [26], or acetic acid with triethylamine [27] rarely methanol, water with the addition of formic acid [28] or formic acid and ammonium acetate [29] were applied. The inhibitory effects on human tumor cell lines: A549, SK-OV-3, SK-MEL-2, and HCT-15 of tubers of *Corydalis ternata* were found [30].

Antiinflammatory, fungitoxic, and apoptosis-inducing activities of compounds from *Dicentra spectabilis* were described [31,32]. The roots of *Dicentra spectabilis* have been used for the treatment of strokes, bruises, improvement of blood circulation. Several alkaloids have been detected from *Dicentra* species: Isocorydine, corydine, dicentrine, protopine, dihydrosanguinarine, sanguinarine, cheilanthifoline, bicuculline, lederine, scoulerine, isoboldine, predicentrine, reticuline, and allocryptopine. The extract from the plant was analysed on C18 column with mobile phase containing acetonitrile, water, ammonium acetate, adjusted to pH 3.0 with acetic acid [33]. The cytotoxicity of compounds from *Dicentra spectabilis* was determined on Raw 264.7 cells [32].

The whole plant of *Meconopsis* species is traditionally used as a Tibetan medicine for the treatment of various diseases, such as inflammation, pain, hepatitis, tuberculosis, headache, and fractures [34,35]. Analysis of alkaloids in extracts obtained from various *Meconopsis* species by HPLC was rarely described and was performed on C18 columns with mobile phases containing acetonitrile, water and ammonia [36] or methanol, water, ammonium acetate, and formic acid [35]. The cytotoxic activity of extracts obtained from *Meconopsis* species was also rarely investigated. Extract from *Meconopsis integrifolia* significantly inhibited human leukemia K562 cell viability, mainly by targeting apoptosis induction and cell cycle arrest in G2/M phase [34]. Extract from *Meconopsis horridula* induced murine leukemia L1210 cell apoptosis and inhibited proliferation through G2/M phase arrest [37].

The aim of this work was to investigate alkaloid compositions by HPLC-DAD and HPLC-MS/MS and anticancer activities of different isoquinoline alkaloids and plant extracts obtained from *Corydalis lutea*, *Dicentra spectabilis*, *Fumaria officinalis*, *Macleaya cordata*, *Mahonia aquifolia*, *Meconopsis cambrica* containing these alkaloids against various cancer cell lines. These extracts have not been previously tested against the cancer cell lines.

## 2. Results and Discussion

### 2.1. HPLC Analysis of Plant Extracts

**Figure 1:**
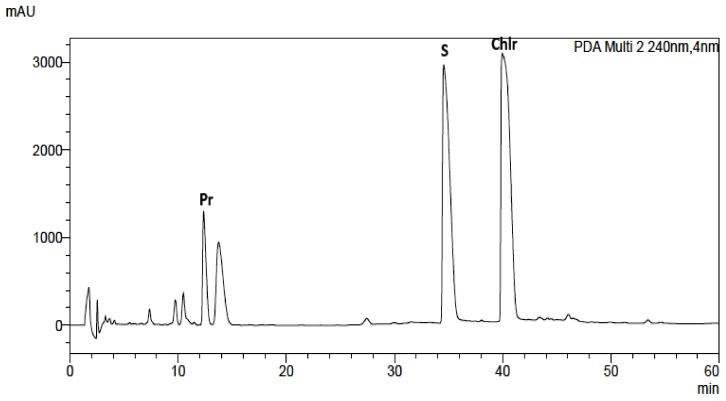
Chromatogram obtained for *Macleaya cordata* leaf extract obtained on Polar RP column with mobile phase containing MeCN, water, and 0.04 ML^−1^ IL. For gradient, see experimental section.

Alkaloid standards (see Table 1) were chromatographed on Hydro RP and Polar RP columns in eluent system containing acetonitrile, water and 0.04 ML^−1^ of 1-butyl-3-methylimidazolium tetrafluoroborate in gradient elution mode described in section “Experimental”. Because on the Hydro RP column with the octadecyl phase there was a worse shape of the peaks, lower theoretical plates number, and poorer separation selectivity of the investigated alkaloids, the RP Polar column was selected for further investigations (Table 1, Figure 1 and Figure S1). Retention times (t_R_), asymmetry factors (As), and theoretical plate number per meter (N/m) for investigated alkaloid standards are presented in Table 1. Application of chromatographic system with double protection against undesirable interactions of analytes with free silanol groups: Phenyl stationary phase with π-π interaction and mobile phase with the addition of ionic liquid as free silanol blocker allow to obtain high system efficiency, symmetrical peaks, and full separation of investigated alkaloids. For all alkaloids, As values between 0.82 and 1.42 and high N/m values (from 33,000 to 700,000) were obtained (Table 1).

The same chromatographic system was used for the analysis of alkaloids in plant extracts obtained from *Corydalis lutea* root and herb, *Dicentra spectabilis*, *Fumaria officinalis*, *Macleaya cordata* leaves and herb, *Mahonia aquifolia* leaves and cortex, *Meconopsis cambrica* root and herb. The presence of alkaloids in plant extracts was identified by comparison of retention times with retention times of alkaloid standards, UV-Vis spectra and additionally confirmed by MS spectra (Figures S11–S21).

The quantitative analysis was performed by a calibration curve method. The number of replicates was three for all concentrations of all alkaloids. Calibration curve equations, correlation coefficients (r), limit of detection (LOD), and limit of quantification (LOQ) obtained for alkaloids are presented in Table 2.

The obtained results also show that the content of alkaloids varied greatly not only with the kind of species but also in different parts of plants (Table 3). The identities of the analyte peaks in plant extracts were confirmed by the comparison of their retention times, UV-Vis spectra with the retention times and spectra of alkaloid standards and by MS detection. An example of the obtained chromatogram is presented in Figure 1. Chromatograms obtained for other plant extracts are presented in Supplementary Materials (Figures S2–S9). MS spectra obtained for alkaloid standards and alkaloids from extracts are presented in Figures S11–S21. Berberine was identified in *Mahonia aquifolium* cortex (above 0.13 mg/g of plant material). Chelerytrine was identified in three investigated extracts obtained from: *Fumaria officinalis*, *Macleaya cordata* leaves, and *Macleaya cordata* herb. A high content of this alkaloid was found in extracts obtained from *Macleaya cordata* especially from leaves (above 5.6 mg/g of plant material). Magnoflorine was determined only in extracts from *Mahonia aquifolium*. The cortex of this plant species contained only 0.086 mg/g of plant material, while in the leaves, the content of magnoflorine was higher than 0.32 mg/g of plant material. Palmatine was identified in *Corydalis lutea* root and herb and *Mahonia aquifolium* cortex extracts. The highest content of this alkaloid was found in the root of *Corydalis lutea*, however, its content was only about 0.1 mg/g of plant material. Protopine was identified in the most investigated extracts. The highest amount of these alkaloids was determined in the extracts obtained from the roots of *Corydalis lutea* and *Dicentra spectabilis*. In both roots, near 5.4 mg of protopine per g of plant material was quantified. Sanquinarine was determined in extracts obtained from *Dicentra spectabilis* herb and root, *Fumaria officinalis*, *Macleaya cordata* leaves and herb, *Meconopsis cambrica* root. Especially a lot of sanguinarine content was found in the leaves of *Macleaya cordata,* above 3 mg/g of plant material. Stylopine was identified in *Corydalis lutea* root and herb and *Fumaria officinalis* extracts. In all plant material, the content of stylopine was above 2 mg/g and in *Corydalis lutea* root was higher than 4 mg/g of plant material.

### 2.2. Investigation of In Vitro Anticancer Activity of Alkaloid Standards

The cytotoxic activity of alkaloid standards: Berberine, chelerythrine, magnoflorine, palmatine, protopine, sanquinarine, and stylopine were carried out using the following human cancer cell lines: Human pharyngeal squamous carcinoma cells (FaDu), human tongue squamous carcinoma cells (SCC-25), human breast adenocarcinoma cell line (MCF-7) and human triple-negative breast adenocarcinoma cell line (MDA-MB-231). The results were expressed as IC50 values, which represent the concentrations that inhibited cell growth by 50% (Table 4).

Varied cytotoxicity of alkaloid standards against the investigated cell lines was determined. The lowest IC_50_ values were obtained for sanquinarine on all tested cell lines (from 0.84 µM against FaDu and MCF-7 to 1.41 µM against SCC-25 cell lines). Very high cytotoxicity against investigated cell lines was also observed for chelerythrine (IC_50_ from 6.11 µM against FaDu to 9.10 µM against MCF-7 cell lines). Low IC_50_ values were found in berberine (IC_50_ from 27.51 against FaDu µM to 113.42 µM against MCF-7 cell lines). Higher IC_50_ values were obtained for the other alkaloids, which indicates their lower cytotoxicity. However, IC_50_ values obtained against all tested cell lines were higher than 500 µM only for magnoflorine.

### 2.3. Investigation of In Vitro Anticancer Activity of Plant Extracts

In vitro cytotoxic activity of the investigated plant extracts was examined on the same cancer cell lines as previously investigated alkaloids, FaDu and SCC-25 cell lines belong to so-called head and neck squamous cell carcinomas (HNSCC), which account for nearly 90% of head and neck cancers. These types of cancers are often resistant to chemotherapy, including even targeted drug therapy [38,39]. Therefore, HNSCCs are characterized by a high recurrence rate and five-year survival rate in patients with HNSCCs is about 40–60%. They are also the eighth most frequent cause of cancer-related deaths. Both FaDu and SCC-25 cell lines are commonly used for testing small molecules and biological samples during cancer drug development [40].

In turn, breast cancer is the most common cancer in women worldwide. The metastasis of cancer cells is the main reason for deaths of patients suffering from breast cancer. Especially the triple-negative breast cancer (TNBC), which is characterized by the lack of expression of HER-2, progesterone (PR) and estrogen (ER) receptors, exhibits poor prognosis. TNBC accounts for 10% of all cases of breast cancers. The five-year survival rate is estimated to be around 62% in TNBC patients and 75% in non-TNBC patients [41]. Taking into consideration the above-mentioned facts, in our current studies, MDA-MB-231, and MCF-7 cells have been investigated as recognized models of TNBC and non-TNBC, respectively [42].

**Figure 2:**
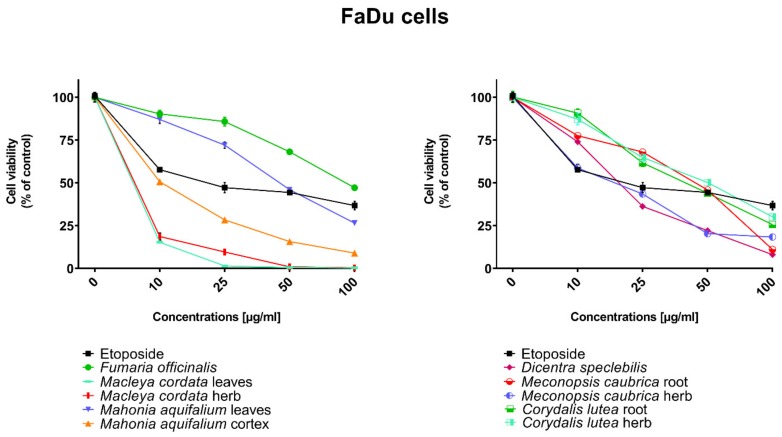
Influence of plant extracts and etoposide concentrations on human pharyngeal squamous carcinoma cells (FaDu) viability.

**Figure 3:**
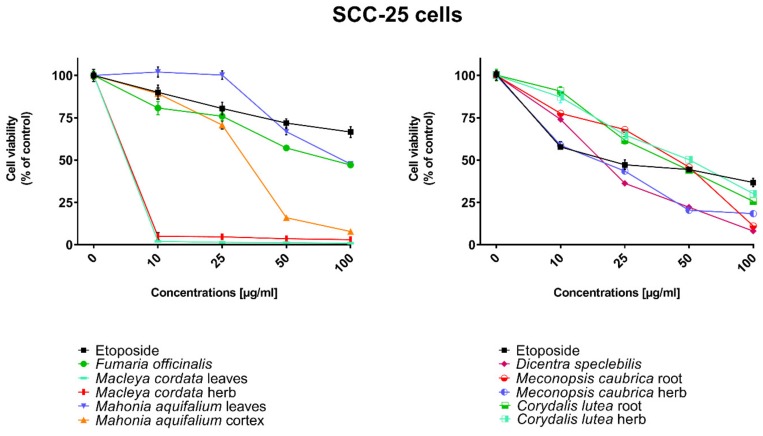
Influence of plant extracts and etoposide concentrations on human tongue squamous carcinoma cells (SCC-25) viability.

**Figure 4:**
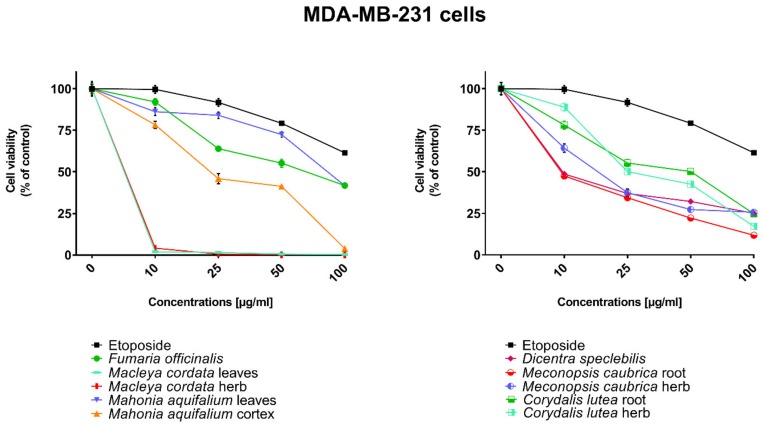
Influence of plant extracts and etoposide concentrations on human triple-negative breast adenocarcinoma cell line (MDA-MB-231) viability.

**Figure 5:**
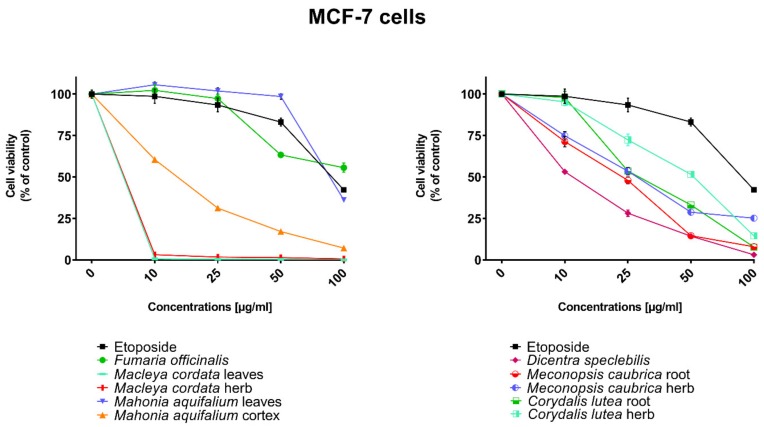
Influence of plant extracts and etoposide concentrations on human breast adenocarcinoma cell line (MCF-7) viability.

All human cancer lines were treated by plant extracts in concentrations 10, 25, 50, and 100 µg/mL for preliminary evaluation of cytotoxic properties of these extracts. For comparison of cytotoxic activity, experiments were also performed for anticancer drug, etoposide, on the same cell lines and at the same concentrations as plant extracts. Results were reported as the percentage of relative viability of the treated cells when compared to the untreated control cells (Figure 2, Figure 3, Figure 4 and Figure 5). All investigated plant extracts exhibit very high cytotoxic activity, especially at higher concentrations. At a concentration of 100 µg/mL, almost all plant extracts showed greater cytotoxic activity against all tested human cancer cell lines compared to cytotoxic activity obtained for etoposide. Lower cytotoxic activity at a concentration of 100 µg/mL was obtained only for the extract from *Fumaria officinalis* against FaDu and MCF-7 cells. Due to the promising results of the preliminary studies, the extract was examined in at least eight different concentrations in order to determine median inhibitory concentration (IC_50_) values (Table 5).

The highest cytotoxic effect on all tested cell lines was observed for extracts obtained from the herb, and especially leaves, of *Macleaya cordata*. The extract obtained from leaves exhibit greater cytotoxic effect on MCF-7, FaDu, and SCC-25 cell lines (viability of cells at the concentration of extracts at 100 µg/mL were 0.06%, 0.33%, and 0.90%, respectively), while the extract obtained from the herb of the plant was more potent against MDA-MB-231 cell lines (viability of cells at concentrations of 100 µg/mL was 0.10%). At a concentration of only 10 µg/mL of the extract obtained from *Macleaya cordata* leaves, the viability of SCC-25 and MDA-MB-231 cell lines was less than 2%. Viability of MCF-7 cell line at a concentration of 10 µg/mL was only 0.66%. This indicated very high cytotoxic activity of the extract, repeatedly greater than the cytotoxicity of etoposide in relation to these cell lines. Moreover, the extract obtained from the herb of this plant showed high cytotoxicity against the same cell lines (cell viability was about or below 5%). IC_50_ values obtained from *Macleaya cordata* leaf extract were very low (in the range of 1.86 µg/mL against MCF-7 cell line–2.19 µg/mL against SCC-25 cell line). IC_50_ values obtained for extracts obtained from *Macleaya cordata* herb were only slightly higher (in the range of 2.14–2.57 µg/mL). In these extracts, a very high content of alkaloids with strong cytotoxic properties against the tested cancer cells was detected. The extract obtained from the leaves contained above 5.6 mg/g of plant material of chelerythrine which is 46.6% of the dry mass of the extract and 3.1 mg/g of plant material of sanquinqrine which is 25.98% of the dry mass of the extract (Table 2). Sanguinarine and chelerythrine have very high cytotoxicity against all tested cancer cell lines. The content of these alkaloids is 72.58% in dry mass of the extract. The plant material seems to be a good candidate for the obtaining of these alkaloids as well as for further research on its anticancer activity. Slightly smaller cytotoxic activity was observed for herb extract obtained from the same plant which corresponds to a lower content of these two alkaloids in this extract (29.42% and 12.83% of chelerythrine and sanguinarine in dry mass of the extract, respectively). These results may also indicate the accumulation of these alkaloids in the leaves of this plant.

Very high cytotoxic activity was also obtained after treating all investigated cell lines by the extract obtained from *Mahonia aquifolium* cortex. The viability of all tested cancer cell lines treated by the extract at a concentration of 100 µg/mL was below 10%. The extract showed the greatest cytotoxic effect on the MDA-MB-231 cell line, the viability was only 3.88%. Significantly lower cytotoxic activity was observed for the extract obtained from *Mahonia aquifolium* leaves. The viability of cells after treating with the extract at a concentration of 100 µg/mL was from 26.5% for FaDu line to 47.63% for SCC-25 cell line. The content of alkaloids in extracts obtained from leaves and cortex of *Mahonia aquifolium* was significantly different. The IC50 values of *Mahonia aquifolium* cortex extract were very low, especially against FaDu and MCF-7 cell lines, 7.67 and 15.71 µg/mL respectively. Higher IC_50_ values were observed for extract from *Mahonia aquifalium* leaves (46.77–97.25 µg/mL). In *Mahonia aquifolium* leaves, only magnoflorine with low cytotoxic activity was determined, but in cortex, besides magnoflorine and palmatine with low and medium cytotoxic activity, also berberine with high cytotoxicity was found. The extract obtained from *Mahonia aquifalium* cortex contained 0.1332 µg/g of plant material of berberine, which is 2.26% of the dry mass of the extract. The extract exhibits the highest cytotoxicity against FaDu cell line. Berberine has also the highest cytotoxic activity against the FaDu cell line. In the extract obtained from *Mahonia aquifalium* leaves, berberine was not identified and cytotoxicity of the extract was also significantly lower. This may indicate a significant effect of berberine content on the cytotoxicity of extracts. Cytotoxicity of the plant extract can be caused by other components of the extract not identified in our investigations. This requires further investigations.

Potential anticancer activity was found for the extract obtained from *Dicentra spectabilis.* The cytotoxic activity of the plant was different from various types of applied cell lines and also significantly changed with the change of extract concentration. Viability of MDA-MB-231 cells were about 25% at the concentration of extract equaled 100 µg/mL. For other cell lines viability of cells treated by *Dicentra spectabilis* extract was significantly lower: 8.04%, 3.11%, and 3.05% for FaDu, MCF-7 and SCC-25 cell lines, respectively. In the plant, the high content of protopine (above 5 mg/g of plant material) and about 0.1 mg/g of plant material of sanquinarine with high cytotoxicity were determined. Extract obtained from *Dicentra spectabilis* exhibits the lowest IC_50_ values against MDA-MB-231 cell line (only 9.66 µg/mL). In the extract, very high content of protopine was found (95.65% of the dry mass of the extract), but protopine exhibits low cytotoxicity against all cell lines tested by us. High cytotoxic activity of the extract can be caused by the presence of high cytotoxic sanguinarine (1.68% of the dry mass of the extract).

High cytotoxic activity against all tested cell lines was also found for the extract obtained from the root of *Meconopsis cambrica.* Viability of cancer cells treated by the extract significantly decreased with the increase of the extract concentration. At a concentration 100 µg/mL, it was 11.82%, 11.10%, 7.98, and 4.40% for MDA-MB-231, FaDu, MCF-7 and SCC-25 cell lines, respectively. The root extract contains sanquinarine having a very strong cytotoxic effect on all cell lines used in the investigations. Less cytotoxicity was found when the extract obtained from the herb of this plant was applied. The lowest viability was obtained for SCC-25 cell line after treating by the extract at a concentration of 100 µg/mL of *Meconopsis cambrica* root. Great differences were obtained in IC_50_ values for extracts from *Meconopsis caubrica* root and herb. For example, IC_50_ for root extract against FaDu cell line was 43.66 µg/mL, while IC_50_ obtained for extracts from the herb was 13.79 µg/mL against the same cell line. Whereas the extract obtained from *Meconopsis caubrica* root exhibits the lowest IC_50_ values against the other cell lines. The cytotoxic properties of the extract may be caused by the presence of very high cytotoxic sanguinarine which is 1.12% and 0.8% of the dry mass of extracts obtained from herb and root, respectively.

Potential anticancer activity was also determined for *Corydalis lutea* root and herb extracts. Higher cytotoxic activity in relation to MCF-7 and FaDu cell lines was observed for the extract obtained from the root in comparison with the extract obtained from the herb, while higher cytotoxicity in relation to SCC-25 and MDA-MB-231 cell lines was obtained for the herb extract in comparison to the extract from root. The highest cytotoxic effect was found when MCF-7 cells were treated by extract obtained from the root at a concentration of 100 µg/mL (viability of cells was only 7.15%). The lowest cytotoxicity was observed for *Corydalis lutea* root against SCC-25 cell line, but at concentrations of 50 and 100 µg/mL was higher than cytotoxic activity of etoposide at the same concentrations. In extracts obtained from this plant alkaloids having the highest cytotoxic activity were not detected. The IC50 values of *Corydalis lutea* root extract to the investigated cell lines were in the range of 29.37–142.14 µg/mL, while the IC50 values of *Corydalis lutea* herb extract in the range of 31.39–49.34 µg/mL. Lower cytotoxic activity of plant extracts obtained from *Corydalis lutea* against the tested cell lines may be the result of a lack of alkaloids, such as chelerythrine, sanguinarine or berberine with high cytotoxicity in these extracts.

The lowest cytotoxic activity against the tested cell lines was found for the extract obtained from *Fumaria officinalis.* The viability of cells after treatment by the extract at a concentration of 100 µg/mL was from 41.74% for MDA-MB-231 cell line to 55.64% for MCF-7 cell line. The extract at the highest concentration showed greater cytotoxic activity in comparison with the cytotoxicity of etoposide against MDA-MB-231 and SCC-25 cell lines., respectively. The highest IC_50_ values from all investigated plant extracts were also obtained for the extract from *Fumaria officinalis* (from 85.60 µg/mL against MDA-MB-231 to >200 µg/mL against. MCF-7 cell line). In this extract, chelerythrine, protopine, sanquinarine, and stylopine were determined, but alkaloids possessing the highest cytotoxic activity (chelerythrine and sanquinarine) were detected in very small concentrations (0.0598 and 0.0097 mg/g of plant material which is 0.49 and 0.08% of the dry mass of the extract).

## 3. Experimental

### 3.1. Chemicals and Plant Material

Acetonitrile (MeCN), methanol (MeOH), 1-butyl-3-methylimidazolium tetrafluoroborate of chromatographic quality were obtained from E. Merck (Darmstadt, Germany), dimethyl sulfoxide (DMSO) was from Sigma-Aldrich (Saint Louis, MO, USA), dimethyl sulfoxide (DMSO) was from Sigma-Aldrich (Saint Louis, MO, USA).

Alkaloid standards (berberine, magnoflorine, palmatine, protopine, sanguinarine, chelerythrine and stylopine) were purchased from Chem Faces Biochemical Co. Ltd. (Wuhan, China). Berberine was purchased from Sigma-Aldrich (St. Louis, MO, USA).

Plant material was collected and identified in the Botanical Garden of Maria Curie-Skłodowska University in Lublin (Poland) in spring and summer 2018.

Plants were divided into roots and aboveground parts. Plants organs were cut into pieces and dried at ambient temperature for one to two weeks.

### 3.2. Apparatus and HPLC Conditions

#### HPLC-DAD

The analysis was performed using an LC-20AD Shimadzu (Shimadzu Corporation, Canby, OR, USA) liquid chromatograph equipped with Synergi Hydro RP 80A (150 × 4.6 mm, 5 μm) and Synergi Polar RP 80A (150 × 4.6 mm, 5 μm) columns. The chromatograph was equipped with a Shimadzu SPD-M20A detector (Shimadzu Corporation, Canby, OR, USA). Detection was carried out at a wavelength of 240 nm. All chromatographic measurements were controlled by a CTO-10ASVP thermostat (Shimadzu Corporation, Canby, OR, USA). The eluent flow rate was 1.0 mL/min. Extracts were injected into the columns using the Rheodyne 20 μL injector. The DAD detector was set in the 200–800 nm range. Data acquisition and processing were carried out with LabSolutions software (Shimadzu Corporation, Kyoto, Japan). The mobile phase was composed of 0.04 ML^−1^ 1-butyl-3-methylimidazolium tetrafluoroborate in water (solvent A) and 1-butyl-3-methylimidazolium tetrafluoroborate in acetonitrile (solvent B) in gradient elution: 0–20 min, 25% B; 20–30 min, 25%–32% B; 30–40 min, 32%–40% B, 40–60 min, 40% B. Flow rate was 1 mL/min.

Calibration curves were constructed by analyzing the alkaloid standards at eight concentrations, ranging from 0.001 to 0.2 mg/mL. The calibration curves were obtained by means of the least square method. The limit of detection (LOD) and limit of quantification (LOQ) obtained for alkaloids were calculated according to the formula: LOD = 3.3 (SD/S), and LOQ = 10 (SD/S), where SD is the standard deviation of response (peak area) and S is the slope of the calibration curve.

HPLC analyses of alkaloid standards and plant extracts were repeated three times.

#### HPLC-MS

Determination of alkaloids was carried out using an HPLC system equipped with the Agilent XDB-C18 1.8 µm 4.6 × 50 mm column. The column was maintained at 20 °C. The injected sample volume was 20 µL, while the mobile phase was composed ACN + 0.1% HCOOOH (30:70) dosed at a flow rate of 0.6 mL/min. The mass spectral analysis was performed on a UHPLC-QTOF/MS model 1260, 6530 Accurate-Mass QTOF LC/MS; Agilent Technologies (Santa Clara, CA, USA) equipped with an ESI interface operating in positive ion mode, with the following set of operation parameters: Capillary voltage, 3500 V; nebulizer pressure, 40 psi; drying gas flow, 7 L/min; drying gas temperature, 295 °C; LC–MS mass spectra were recorded across the range mass range 40–370 m/z; fragmentor 195 V. The HPLC–MS data were acquired and quantified with the use of MassHunter Workstation software. The data were further processed using Microsoft Excel. The instrument was operated in selected ions monitoring mode (SIM) and multiple reaction monitoring (MRM) as well. The monitored pseudomolecular ions [M^+^H]^+^ are presented in Table 6.

### 3.3. Extraction Procedure

The previously described procedure of alkaloids extraction from plant material was applied after minor modifications [43,44].

Samples (5 g) of each plant were macerated with 100 mL ethanol for 72 h and continuously extracted in an ultrasonic bath for 5 h. Extracts were filtered, the solvent evaporated under vacuum, and the residues dissolved in 30 mL of 2% sulfuric acid and defatted with diethyl ether (3 × 40 mL). The aqueous layers were subsequently basified with 25% ammonia to a pH of 9.5–10 and the alkaloids extracted with chloroform (3 × 50 mL). After evaporation of the organic solvent, the dried extracts were dissolved in 5 mL MeOH prior to HPLC analysis. Recovery (%) obtained for alkaloids by the extraction procedure is presented in Table S1.

### 3.4. Investigation of Cytotoxic Activity

Cytotoxic properties of the tested plant extracts and respective secondary metabolites’ standards were examined using human pharyngeal squamous carcinoma cells (FaDu), human tongue squamous carcinoma cells (SCC-25), human breast adenocarcinoma cell line (MCF-7), human triple-negative breast adenocarcinoma cell line (MDA-MB-231). Cell lines used in experiments were obtained from the American Type Culture Collection (ATCC, Manassas, VA, USA). All media, buffers, nutrients, and solutions necessary for cell culturing were from Sigma-Aldrich (St. Louis, MO, USA). FaDu cells were cultured using Eagle’s minimum essential medium (MEM) supplemented with 10% fetal bovine serum, 100 U/mL of penicillin, and 100 mg/mL of streptomycin. SCC-25 was cultured in Dulbecco’s modified Eagle’s medium/nutrient mixture F-12 Ham (DMEM/F12) supplemented with 10% fetal bovine serum, 400 ng/mL hydrocortisone, 100 U/mL of penicillin, and 100 mg/mL of streptomycin (all from Sigma-Aldrich). MCF-7 and MDA-MB-231 cells were cultured using Dulbecco’s modified Eagle’s medium-high glucose (DMEM) containing 10% fetal bovine serum, 100 U/mL of penicillin and 100 mg/mL of streptomycin. Cells were maintained at 37 °C in a 5% CO_2_ atmosphere. Both the dried plant extracts and standards were dissolved in DMSO in order to obtain stock solutions at a concentration of 50 mg/mL. On the day of the experiment, suspension of cells (1 × 105 cells/mL) in the respective medium containing 10% FBS was applied to a 96-well plate at 100μL per well. After 24 h of incubation, the medium was removed from wells and replaced by different concentrations (10–100 µg/mL) of plant extracts or standards in a medium containing 2% FBS. Control cells were cultured only with a medium containing 2% FBS. Cytotoxicity of DMSO was also checked at concentrations present in respective dilutions of stock solutions. After 24 h incubation, 15 μL of MTT working solution (5mg/mL in PBS) was added to each well. The plate was incubated for 3 h. Subsequently, 100 uL of 10% SDS solution was added to each well. Cells were incubated overnight at 37 °C to dissolve the precipitated formazan crystals. The concentration of the dissolved formazan was evaluated by measuring the absorbance at λ = 570 nm using a microplate reader (Epoch, BioTek Instruments, Inc., USA). Two independent experiments were performed in triplicate. The viability of cells incubated with the increased concentrations of plant extracts was expressed as % of the viability of control (untreated) cells (Figure 2, Figure 3, Figure 4 and Figure 5). The investigated standards and extracts were subsequently assayed in at least eight different concentrations in order to calculate the respective IC_50_ from the logarithmic dose-response curve (Table 4 and Table 5). The results of the MTT assay were expressed as mean ± SD. DMSO in the concentrations present in the dilutions of stock solutions did not influence the viability of the tested cells.

## 4. Conclusions

Our studies confirmed a strong ability of the examined alkaloids to inhibit proliferation of cancer cells. The highest cytotoxic activity against all tested cancer cell lines was observed for sanguinarine (IC_50_ < 1.5 µM). Very high cytotoxicity was also obtained for chelerythrine (IC_50_ < 10 µM against all investigated cell lines).

Our data demonstrated that the extracts obtained from *Corydalis lutea* root and herb, *Dicentra spectabilis*, *Fumaria officinalis*, *Macleaya cordata* leaves and herb, *Mahonia aquifolia* leaves and cortex, *Meconopsis cambrica* root and herb have very high cytotoxic activity against MDA-MB-231, FaDu, MCF-7 and SCC-25 cancer cell lines. To the best of our knowledge, the cytotoxic activity of the extracts has not been yet investigated against the cell lines tested by us.

Almost all investigated extracts, especially at higher concentrations, showed cytotoxic activity against tested cell lines significantly higher than cytotoxic activity of an anticancer drug, etoposide.

Higher cytotoxicity was found for extracts containing highly cytotoxic alkaloids: chelerythrine, sanguinarine, and berberine. The highest cytotoxic activity against all tested cancer cell lines (IC_50_ were between 1.86 to 2.19 µg/mL) was observed after applying the *Macleaya cordata* leaf extract containing 46.6 % of the dry mass of the extract of chelerythrine, and 25.98% of sanquinarine.

The differences between the cytotoxic activities of the different parts of investigated plants strongly depend on alkaloids content and a synergic effect of the different alkaloids may influence on extract activities.

The investigated plant material, especially that obtained from *Macleaya cordata*, *Mahonia aquifalium*, *Dicentra speclebilis* and *Meconopsis caubrica,* seems to be promising for further research on its anticancer activity.

## Figures and Tables

**Table 1:** Values of retention time (t_R_), asymmetry factor (A_S_), and theoretical plate number per meter (N/m) obtained for alkaloid standards.

Name of Compound	Hydro RP (Octadecyl Stationary Phase)			Polar RP (Phenyl Stationary Phase)			
	t_R_	As	N/m		t_R_	As	N/m
Berberine	23.57	0.72	33650		34.74	1.42	243280
Chelerythrine	32.29	0.82	190010		40.75	1.37	708700
Magnoflorine	3.12	0.84	8780		3.89	0.82	33190
Palmatine	19.70	*	*		29.78	1.18	126770
Protopine	9.04	0.68	30160		12.54	0.97	49630
Sanquinarine	19.07	*	*		34.87	1.01	413350
Stylopine	13.01	1.28	58300		19.36	0.98	355570

* Very asymmetrical peak.

**Table 2:** Equation of calibration curve, correlation coefficients (r), limit of detection (LOD) and limit of quantification (LOQ) values.

**Alkaloid**	**Equation of Calibration Curve**	**r**	**LOD** **[mg/mL]**	**LOQ** **[mg/mL]**
Berberine	*y* = 72178227*x* − 370170	0.9973	0.0151	0.0457
Chelerythrine	*y* = 84228691*x* + 413980	0.9998	0.0040	0.0123
Magnoflorine	*y* = 23972503*x* + 263324	0.9992	0.0094	0.0287
Palmatine	*y* = 51166752*x* + 511129	0.9991	0.0108	0.0327
Protopine	*y* = 7344826*x* + 64160	0.9992	0.0095	0.0288
Sanguinarine	*y* = 80589787*x* + 606317	0.9997	0.0123	0.0371
Stylopine	*y* = 879342*x* − 13994	0.9996	0.0241	0.0729

**Table 3:** Content of alkaloids in plant samples.

Name of Compound	Content of Alkaloids (mg/g of Plant Material)									
	*Corydalis lutea* Root	*Corydalis lutea* Herb	*Dicentra speclebilis*	*Fumaria officinalis*	*Macleya cordata* Leaves	*Macleya cordata* Herb	*Mahonia aquifalium* Cortex	*Mahonia aquifalium* Leaves	*Meconopsis cambrica* Root	*Meconopsis cambrica* Herb
Berberine	ND	ND	ND	ND	ND	ND	0.1332	ND	ND	ND
Chelerythrine	ND	ND	ND	0.0598	5.6061	1.7654	ND	ND	ND	ND
Magnoflorine	ND	ND	ND	ND	ND	ND	0.0863	0.3251	ND	ND
Palmatine	0.1041	0.03168	ND	ND	ND	ND	0.0360	ND	ND	ND
Protopine	5.4562	0.5526	5.3756	2.7873	1.7621	0.4731	ND	ND	0.0236	0.0787
Sanquinarine	ND	ND	0.0940	0.0097	3.1253	0.7699	ND	ND	0.0504	0.0542
Stylopine	4.0774	2.0725	ND	2.8251	ND	ND	ND	ND	ND	ND

ND, not detected.

**Table 4:** Cytotoxic effect of the investigated alkaloids and etoposide against FaDu, SCC-25, MCF-7, and MDA-MB-231 cells.

	IC_50_ [µM] ± SD			
	FaDu	SCC 25	MCF-7	MDA-MB-231
Berberine	27.51± 6.72	84.24 ± 7.75	113.42 ± 14.69	51.05 ± 9.07
Chelerythrine	6.11 ± 0.32	7.49 ± 0.77	9.10 ± 0.55	7.11 ± 0.26
Magnoflorine	>500	>500	>500	>500
Palmatine	94.27 ± 9.39	320.23 ± 46.34	454.77 ± 24.52	423.38 ± 34.22
Protopine	234.95 ± 37.55	298.73 ± 33.42	429.54 ± 34.92	370.13 ± 22.18
Sanguinarine	0.84 ± 0.03	1.41 ± 0.12	0.84 ± 0.06	1.26 ± 0.03
Stylopine	193.26 ± 3.80	340.40 ± 31.21	207.18 ± 16.95	489.51 ± 40.86
Etoposide	38.73 ± 1.56	223.94 ± 24.81	136.48 ± 8.95	219.31 ± 24.47

**Table 5:** Cytotoxic activity of the investigated plant extracts against different cancer cell lines.

	IC_50_ [µg/mL] ± SD			
Plant Sample	FaDu	SCC-25	MCF-7	MDA-MB-231
*Fumaria officinalis*	102.76 ± 13.03	101.46 ± 5.96	>200	85.60 ± 13.25
*Macleaya cordata* leaves	1.94 ± 0.27	2.19 ± 0.09	1.86 ± 0.08	2.09 ±0.10
*Macleaya cordata* herb	2.57 ± 0.24	2.50 ± 0.26	2.14 ± 0.18	2.42 ± 0.21
*Mahonia aquifalium* leaves	46.77 ± 7.84	97.25 ± 8.07	89.14 ± 2.73	90.71 ± 7.29
*Mahonia aquifalium* cortex	7.67 ± 0.82	31.37 ± 2.29	15.71 ± 1.92	31.87 ± 4.35
*Dicentra speclebilis*	19.88 ± 2.26	29.55 ± 4.09	11.66 ± 1.36	9.66 ± 0.42
*Meconopsis caubrica* root	43.66 ± 4.78	27.96 ± 1.03	23.26 ± 3.69	9.98 ± 1.34
*Meconopsis caubrica* herb	13.70 ± 1.24	48.02 ± 4.89	31.60 ± 2.42	21.10 ± 1.96
*Corydalis lutea* root	38.08 ± 3.50	142.14 ± 10.58	29.37 ± 4.01	57.98 ± 10.67
*Corydalis lutea* herb	47.47 ± 4.50	48.06 ± 0.86	49.34 ± 5.05	31.39 ± 1.82
Etoposide	22.80 ± 0.92	131.80 ± 14.6	80.33 ± 5.27	129.08 ± 14.4

**Table 6:** The monitored pseudomolecular ions [M^+^H]^+^ parameters.

	m/z (~)	Q1 (~)	Q3 (~)	Iso. Width	Collison Energy
Berberine	336	320	292	Medium(~4m/z)	35
Chelerythrine	348	332	304	Medium(~4m/z)	35
Magnoflorine	342	296	236	Medium(~4m/z)	30
Palmatine	352	336	308	Medium(~4m/z)	25
Protopine	354	189	149	Medium(~4m/z)	35
Sanguinarine	332	273	316	Medium(~4m/z)	25
Stylopine	324	176	149	Medium(~4m/z)	35

## Supplementary Materials

The following are available online at https://www.mdpi.com/2072-6651/11/10/575/s1, Figure S1A. Chromatogram obtained for alkaloid standards obtained on Hydro RP column with mobile phase containing MeCN, water and 0.04 ML-1 IL. Gradient see experimental section, Figure S1B. Chromatogram obtained for alkaloid standards obtained on Polar RP column with mobile phase containing MeCN, water and 0.04 ML-1 IL. Gradient see experimental section, Figure S2. Chromatogram obtained for Meconopsis caubrica root extract obtained on Polar RP column with mobile phase containing MeCN, water and 0.04 ML-1 IL. Gradient see experimental section, Figure S3. Chromatogram obtained for Meconopsis caubrica herb extract obtained on Polar RP column with mobile phase containing MeCN, water and 0.04 ML-1 IL. Gradient see experimental section, Figure S4. Chromatogram obtained for *Mahonia aquifalium* leaves extract obtained on Polar RP column with mobile phase containing MeCN, water and 0.04 ML-1 IL. Gradient see experimental section, Figure S5. Chromatogram obtained for *Mahonia aquifalium* cortex extract obtained on Polar RP column with mobile phase containing MeCN, water and 0.04 ML-1 IL. Gradient see experimental section, Figure S6. Chromatogram obtained for *Macleaya cordata* herb extract obtained on Polar RP column with mobile phase containing MeCN, water and 0.04 ML-1 IL. Gradient see experimental section, Figure S7. Chromatogram obtained for Dicentra speclebilis extract obtained on Polar RP column with mobile phase containing MeCN, water and 0.04 ML-1 IL. Gradient see experimental section, Figure S8. Chromatogram obtained for *Fumaria officinalis* extract obtained on Polar RP column with mobile phase containing MeCN, water and 0.04 ML-1 IL. Gradient see experimental section, Figure S9. Chromatogram obtained for *Corydalis lutea* root extract obtained on Polar RP column with mobile phase containing MeCN, water and 0.04 ML-1 IL. Gradient see experimental section, Figure S10. Chromatogram obtained for *Corydalis lutea* herb extract obtained on Polar RP column with mobile phase containing MeCN, water and 0.04 ML-1 IL. Gradient see experimental section, Figure S11A. MS spectrum obtained for standard of berberine, Figure S11B. MS spectrum obtained for standard of chelerythrine, Figure S11C. MS spectrum obtained for standard of magnoflorine, Figure S11D. MS spectrum obtained for standard of palmatine, Figure S11E. MS spectrum obtained for standard of protropine, Figure S11F. MS spectrum obtained for standard of sanguinarine, Figure S11G. MS spectrum obtained for standard of stylopine, Figure S12A. MS spectrum obtained for *Mahonia aquifalium* cortex extract, Figure S12B. MS spectrum obtained for berberine from *Mahonia aquifalium* cortex extract, Figure S12C. MS spectrum obtained for palmatine from *Mahonia aquifalium* cortex extract, Figure S12D. MS spectrum obtained for magnoflorine from *Mahonia aquifalium* cortex extract, Figure S13A. MS spectrum obtained for *Mahonia aquifalium* leaves, Figure S13B. MS spectrum obtained for magnoflorine from *Mahonia aquifalium* leaves, Figure S14A. MS spectrum obtained for *Fumaria officinalis* extract, Figure S14B. MS spectrum obtained for chelerythrine from *Fumaria officinalis* extract, Figure S14C. MS spectrum obtained for protopine from *Fumaria officinalis* extract, Figure S14D. MS spectrum obtained for sanguinarine from *Fumaria officinalis* extract, Figure S14E. MS spectrum obtained for stylopine from *Fumaria officinalis* extract, Figure S15A. MS spectrum obtained for *Macleaya cordata* leaves extract, Figure S15B. MS spectrum obtained for chelerythrine from *Macleaya cordata* leaves extract, Figure S15C. MS spectrum obtained for protopine from *Macleaya cordata* leaves extract, Figure S15D. MS spectrum obtained for sanguinarine from *Macleaya cordata* leaves extract, Figure S16A. MS spectrum obtained for chelerythrine from *Macleaya cordata* herb extract, Figure S16B. MS spectrum obtained for chelerythrine from *Macleaya cordata* herb extract, Figure S16C. MS spectrum obtained for protopine from *Macleaya cordata* herb extract, Figure S16D. MS spectrum obtained for sanguinarine from *Macleaya cordata* herb extract, Figure S17A. MS spectrum obtained for *Corydalis lutea* root extract, Figure S17B. MS spectrum obtained for palmatine from *Corydalis lutea* root extract, Figure S17C. MS spectrum obtained for protopine from *Corydalis lutea* root extract, Figure S17D. MS spectrum obtained for stylopine from *Corydalis lutea* root extract, Figure S18A. MS spectrum obtained for *Corydalis lutea* herb extract, Figure S18B. MS spectrum obtained for palmatine from *Corydalis lutea* herb extract, Figure S18C. MS spectrum obtained for protopine from *Corydalis lutea* herb extract, Figure S18D. MS spectrum obtained for stylopine from *Corydalis lutea* herb extract, Figure S19A. MS spectrum obtained for *Dicentra spectabilis* herb extract, Figure S19B. MS spectrum obtained for protopine from *Dicentra spectabilis* herb extract, Figure S19C. MS spectrum obtained for sanguinarine from *Dicentra spectabilis* herb extract, Figure S20A. MS spectrum obtained for *Meconopsis cambrica* root extract, Figure S20B. MS spectrum obtained for protopine from *Meconopsis cambrica* root extract, Figure S20C. MS spectrum obtained for sanguinarinee from *Meconopsis cambrica* root extract, Figure S21A. MS spectrum obtained for *Meconopsis cambrica* herb extract, Figure S21B. MS spectrum obtained for protopine from *Meconopsis cambrica* herb extract, Table S1. Exemplary of recoveries of extraction (%) obtained for alkaloids.
