# Supplementary material for: Determination of Selected Isoquinoline Alkaloids from Mahonia aquifolia; Meconopsis cambrica; Corydalis lutea; Dicentra spectabilis; Fumaria officinalis; Macleaya cordata Extracts by HPLC-DAD and Comparison of Their Cytotoxic Activity
Source: Toxins (Basel). 2019 Oct 2;11(10):575. doi: 10.3390/toxins11100575 (PMC6832497; doi:10.3390/toxins11100575)
Supplement: Supplementary file 1 [file toxins-11-00575-s001.pdf]

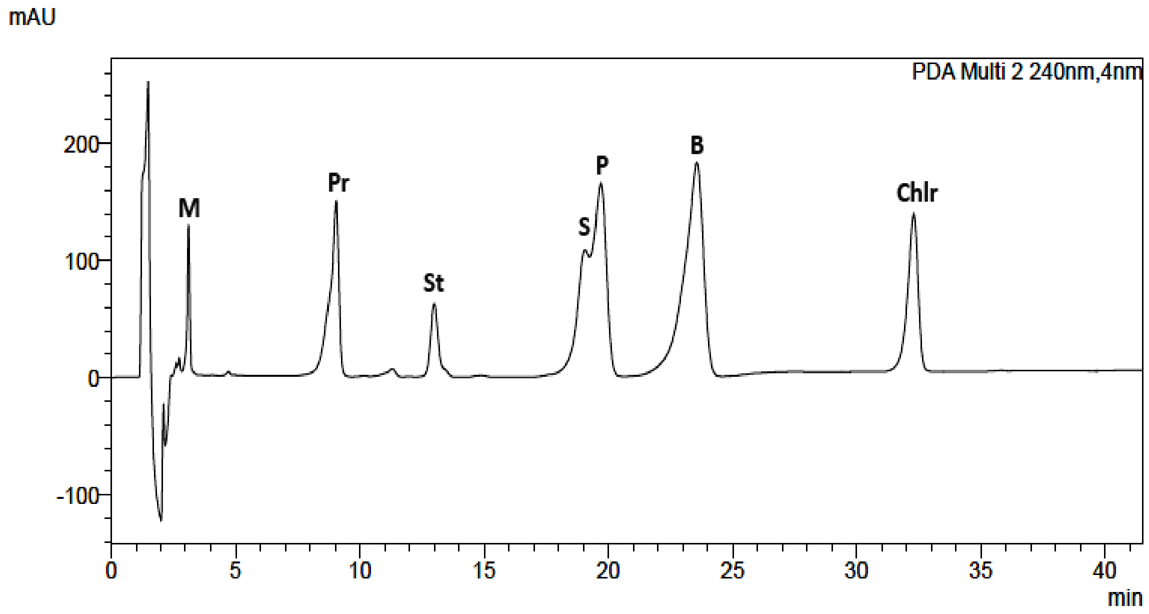

Figure S1A. Chromatogram obtained for alkaloid standards obtained on Hydro RP column with mobile phase containing MeCN, water and 0.04 ML-1 IL. Gradient see experimental section.

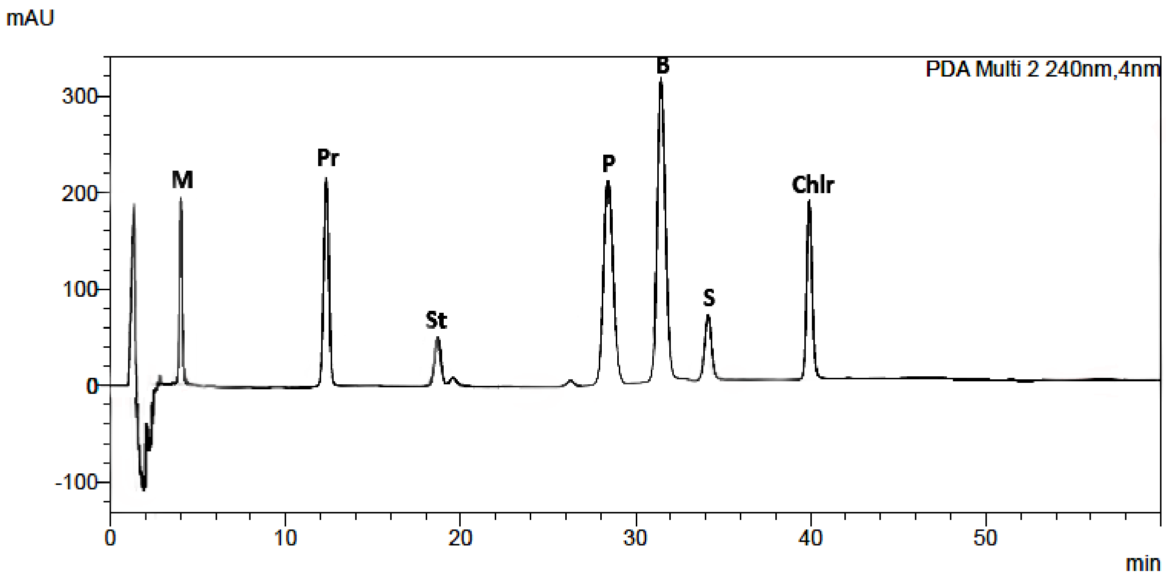

Figure S1B. Chromatogram obtained for alkaloid standards obtained on Polar RP column with mobile phase containing MeCN, water and 0.04 ML-1 IL. Gradient see experimental section.

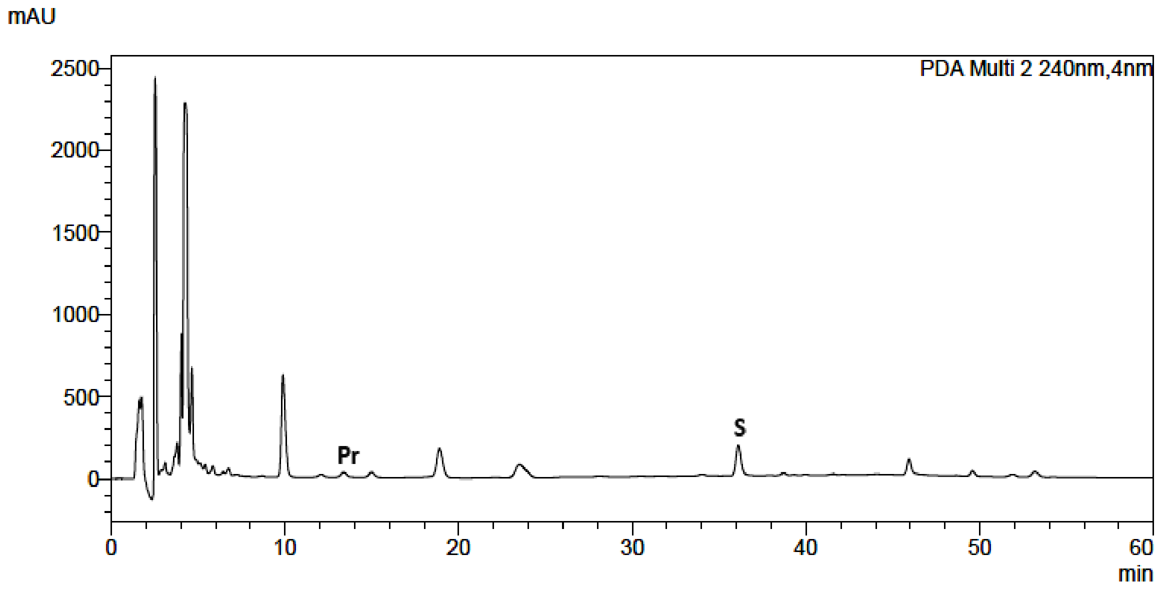

Figure S2. Chromatogram obtained for *Meconopsis caubrica* root extract obtained on Polar RP column with mobile phase containing MeCN, water and 0.04 ML-1 IL. Gradient see experimental section.

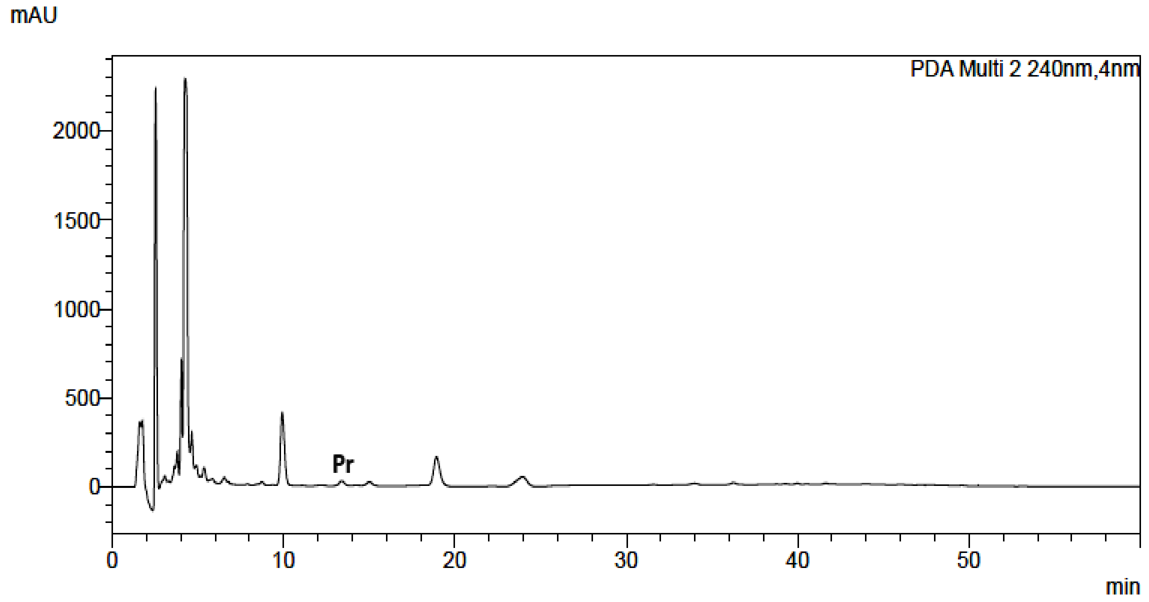

Figure S3. Chromatogram obtained for *Meconopsis caubrica* herb extract obtained on Polar RP column with mobile phase containing MeCN, water and 0.04 ML-1 IL. Gradient see experimental section.

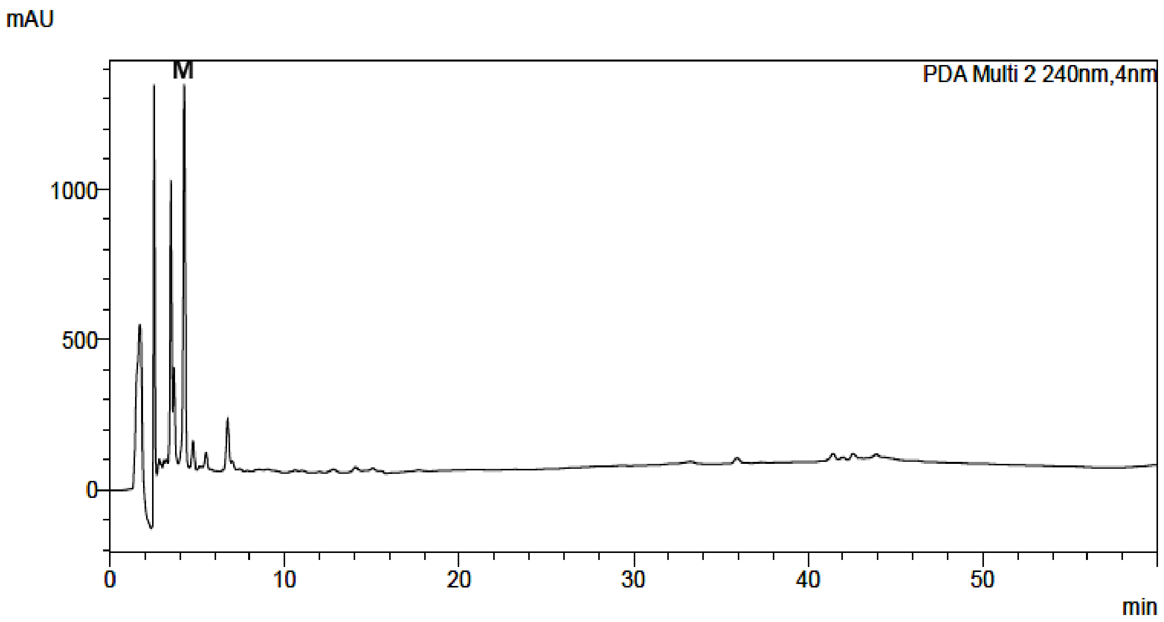

Figure S4. Chromatogram obtained for *Mahonia aquifalium* leaves extract obtained on Polar RP column with mobile phase containing MeCN, water and 0.04 ML-1 IL. Gradient see experimental section.

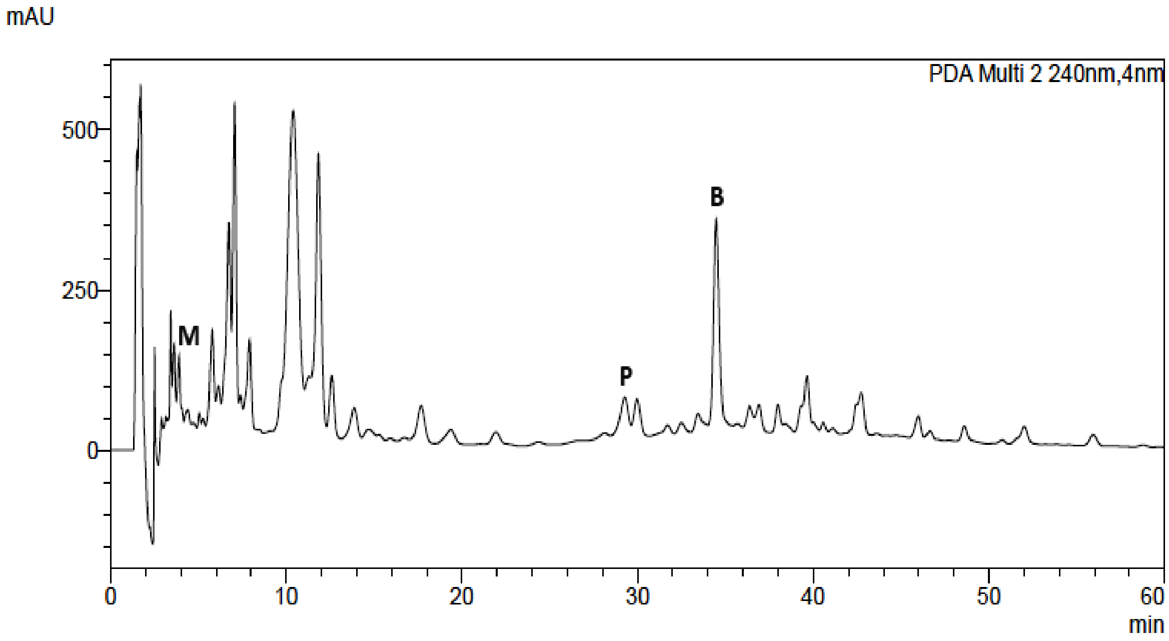

Figure S5. Chromatogram obtained for *Mahonia aquifalium* cortex extract obtained on Polar RP column with mobile phase containing MeCN, water and 0.04 ML-1 IL. Gradient see experimental section.

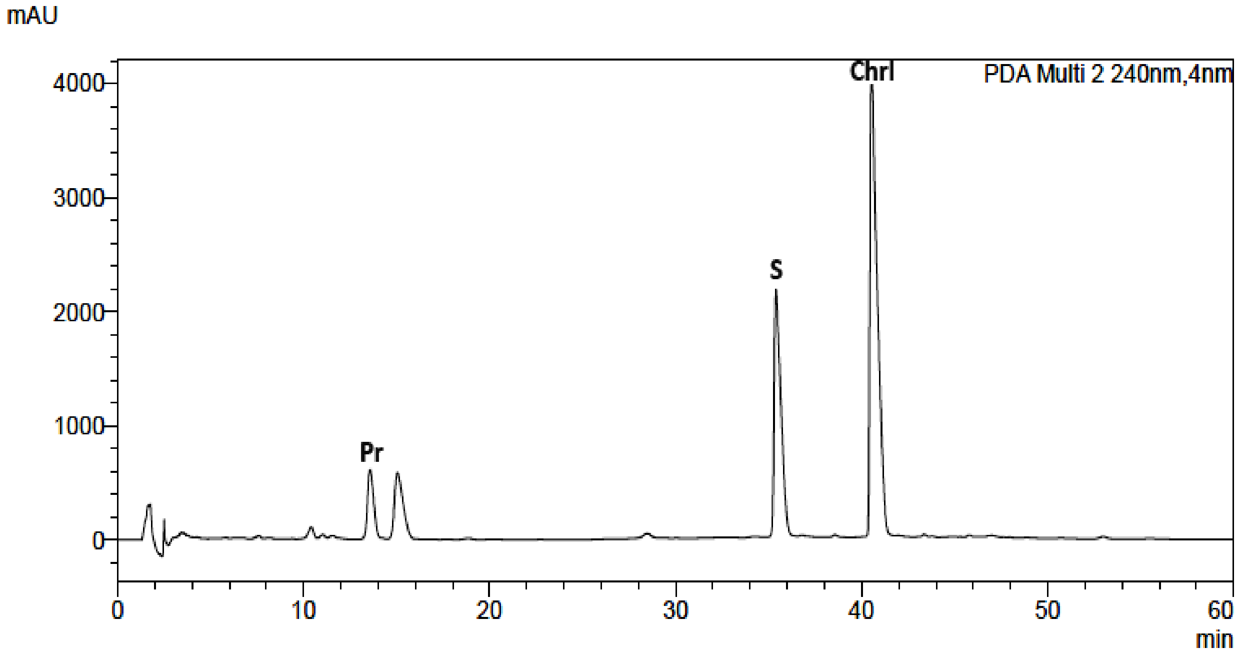

Figure S6. Chromatogram obtained for *Macleaya cordata* herb extract obtained on Polar RP column with mobile phase containing MeCN, water and 0.04 ML-1 IL. Gradient see experimental section.

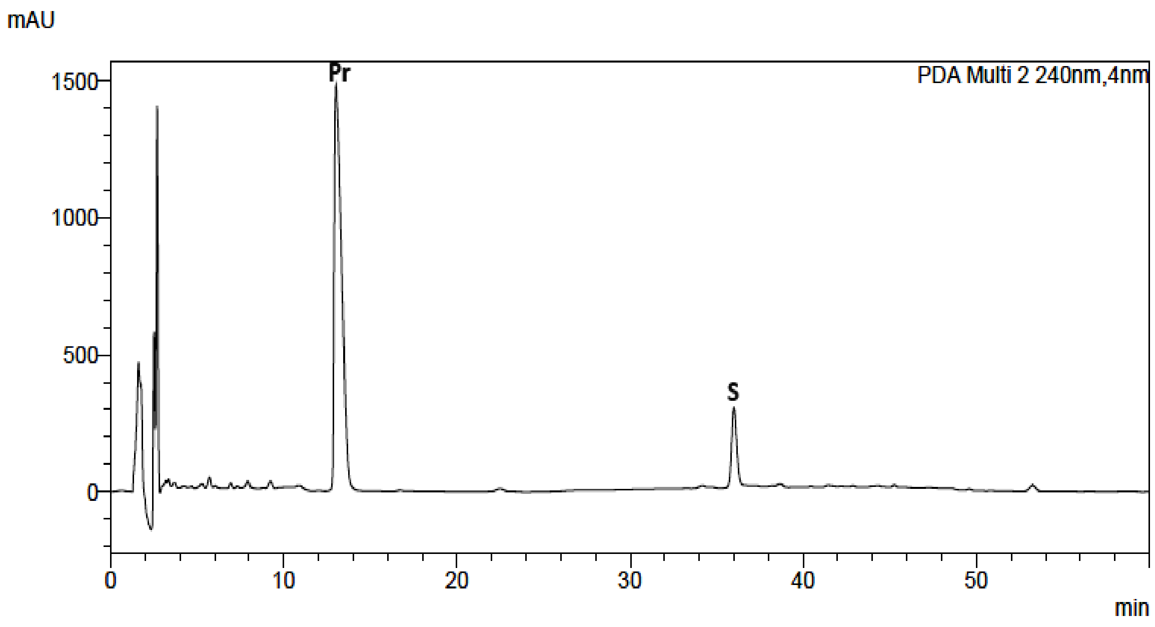

Figure S7. Chromatogram obtained for *Dicentra speclebilis* extract obtained on Polar RP column with mobile phase containing MeCN, water and 0.04 ML-1 IL. Gradient see experimental section.

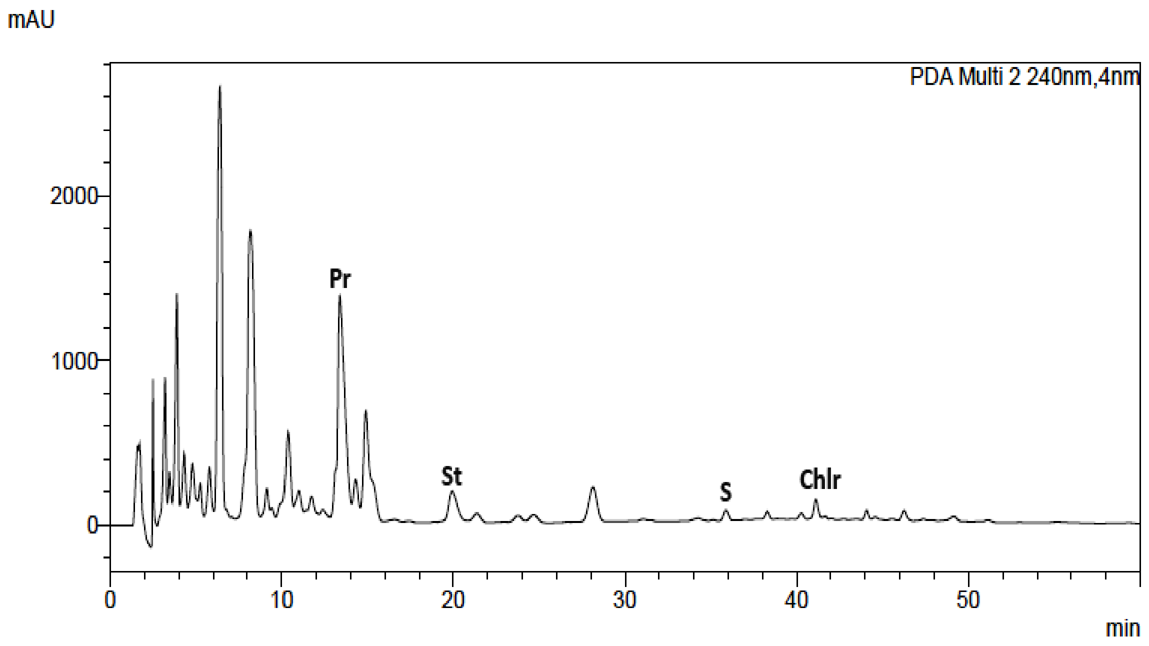

Figure S8. Chromatogram obtained for *Fumaria officinalis* extract obtained on Polar RP column with mobile phase containing MeCN, water and 0.04 ML-1 IL. Gradient see experimental section.

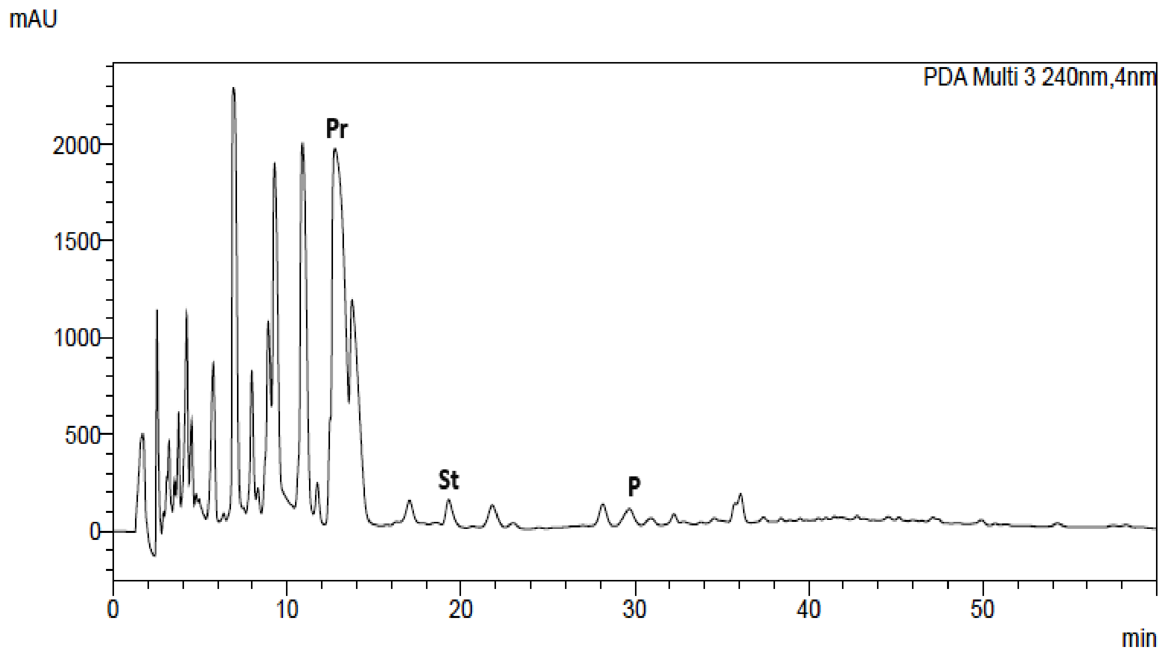

Figure S9. Chromatogram obtained for *Corydalis lutea* root extract obtained on Polar RP column with mobile phase containing MeCN, water and 0.04 ML-1 IL. Gradient see experimental section.

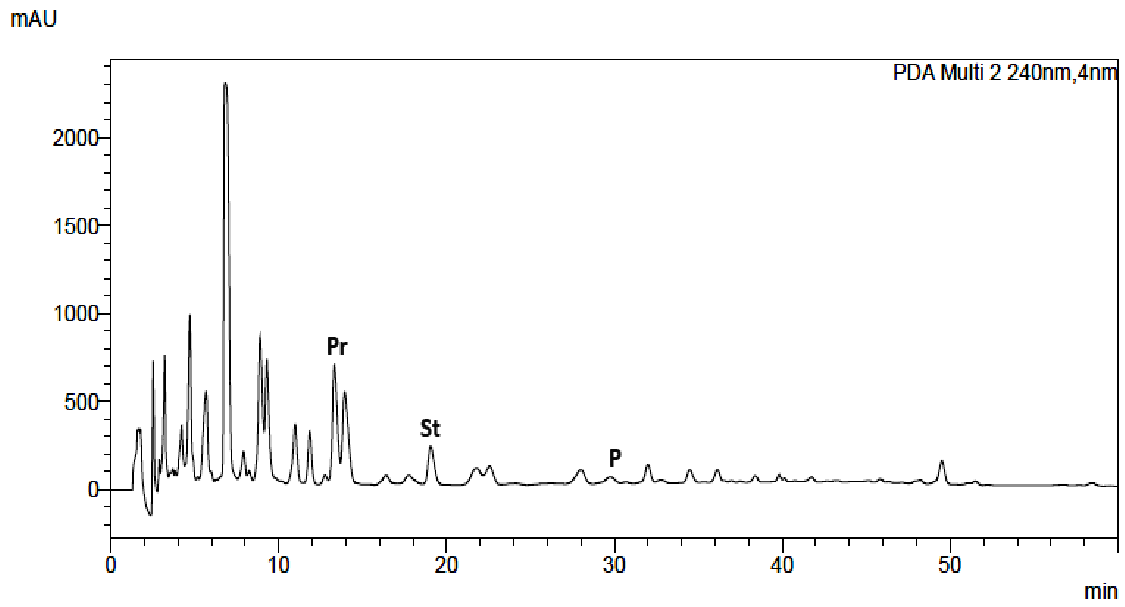

Figure S10. Chromatogram obtained for *Corydalis lutea* herb extract obtained on Polar RP column with mobile phase containing MeCN, water and 0.04 ML-1 IL. Gradient see experimental section.

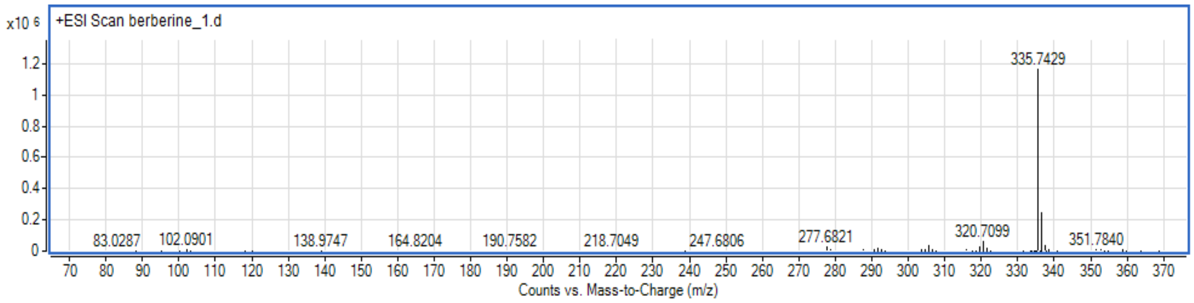

Figure S11A. MS spectrum obtained for standard of berberine.

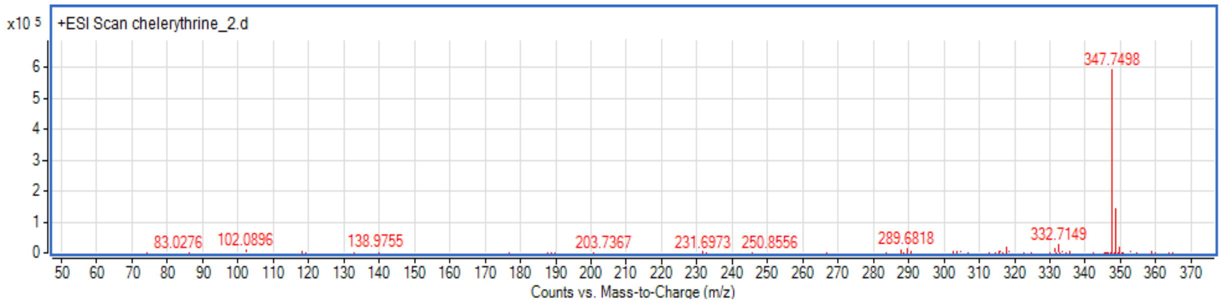

Figure S11B. MS spectrum obtained for standard of chelerythrine.

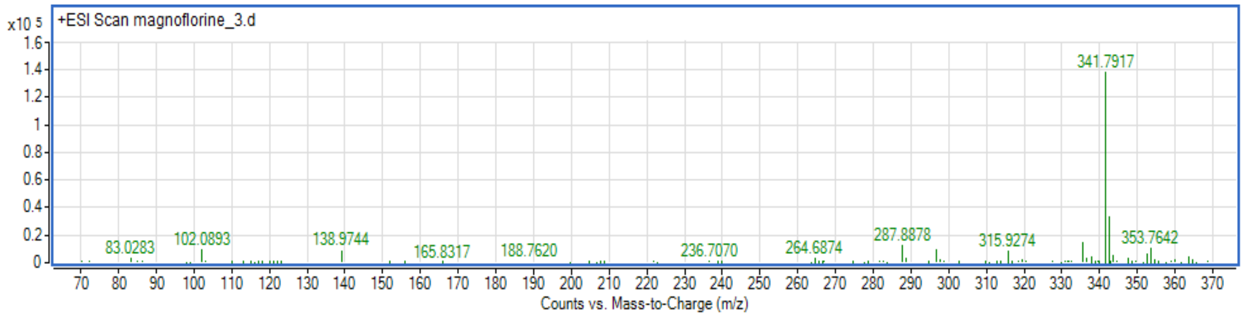

Figure S11C. MS spectrum obtained for standard of magnoflorine.

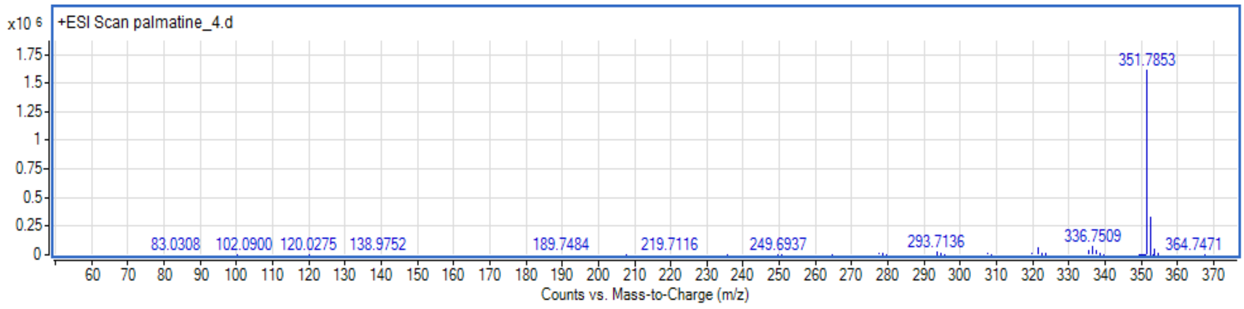

Figure S11D. MS spectrum obtained for standard of palmatine.

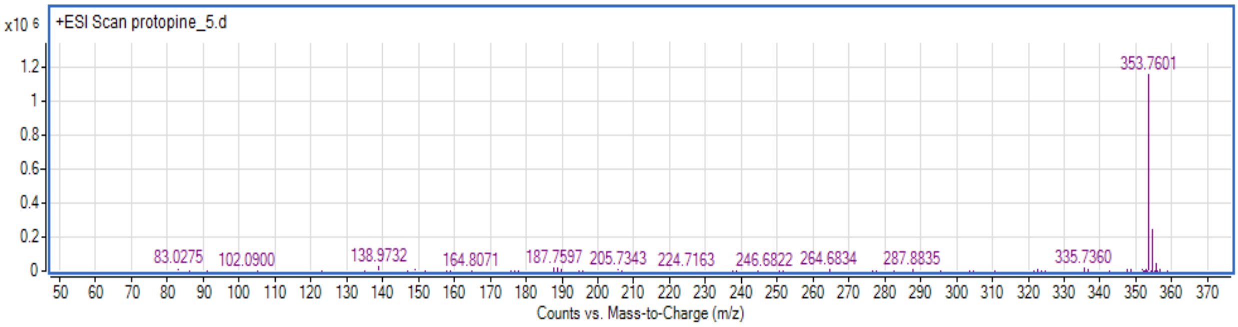

Figure S11E. MS spectrum obtained for standard of protropine.

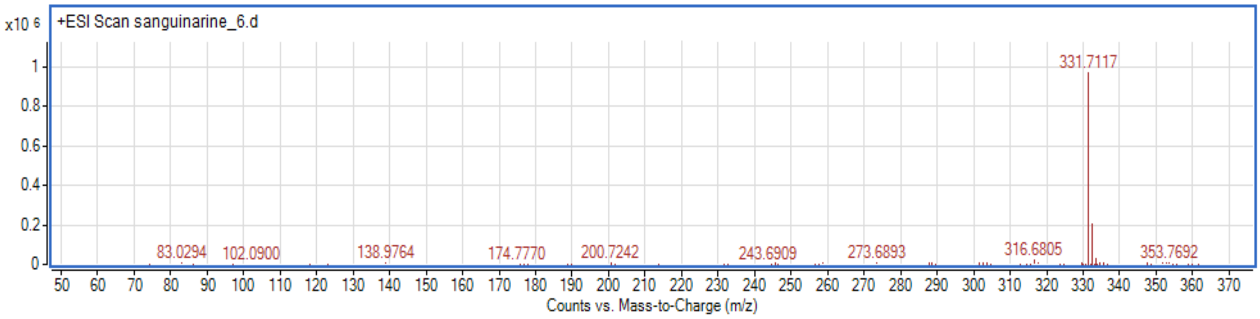

Figure S11F. MS spectrum obtained for standard of sanguinarine.

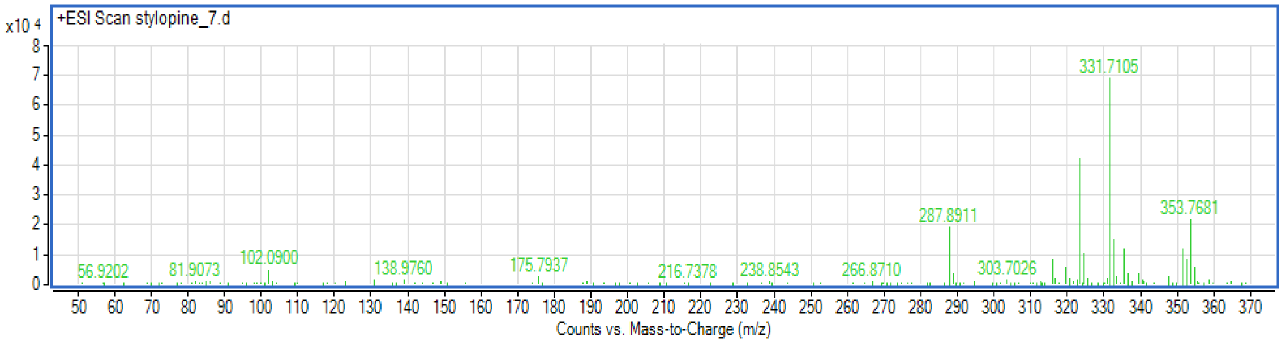

Figure S11G. MS spectrum obtained for standard of stylopine.

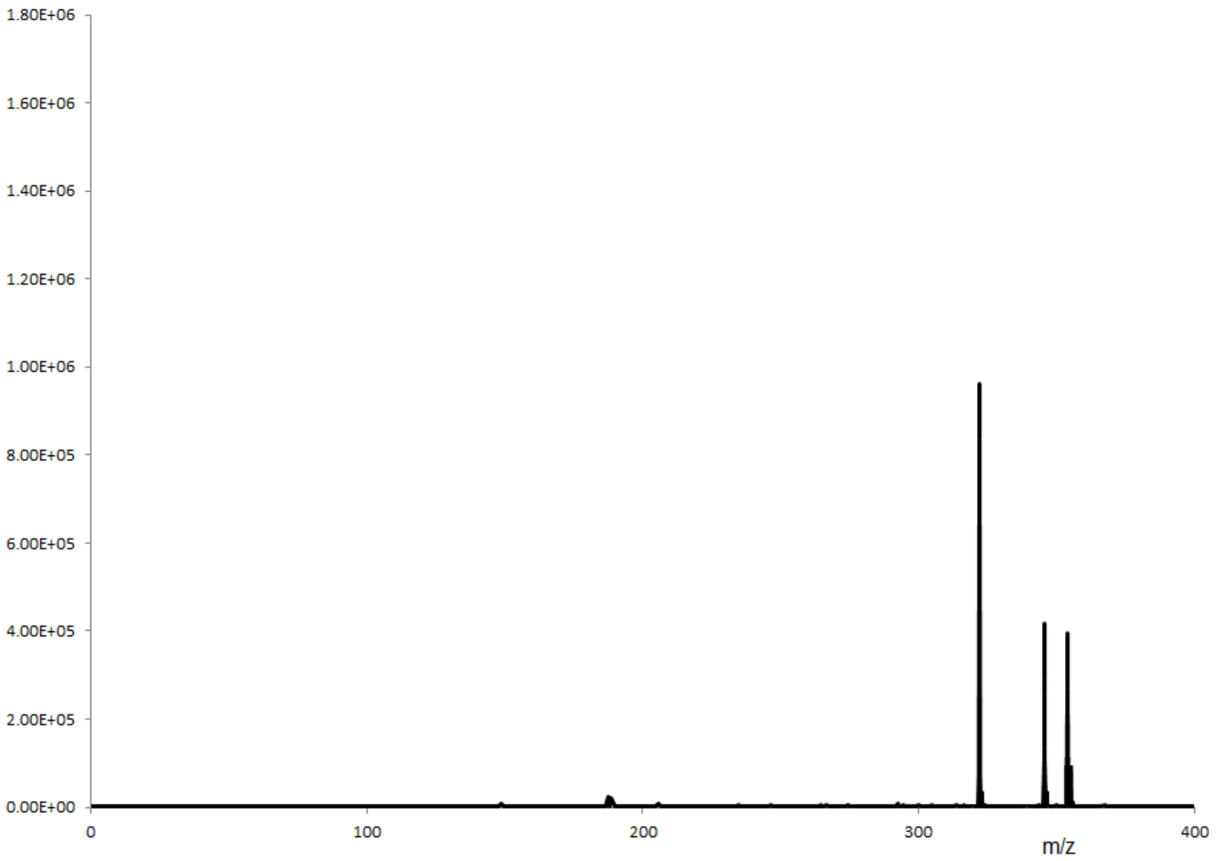

Figure S12A. MS spectrum obtained for *Mahonia aquifalium* cortex extract.

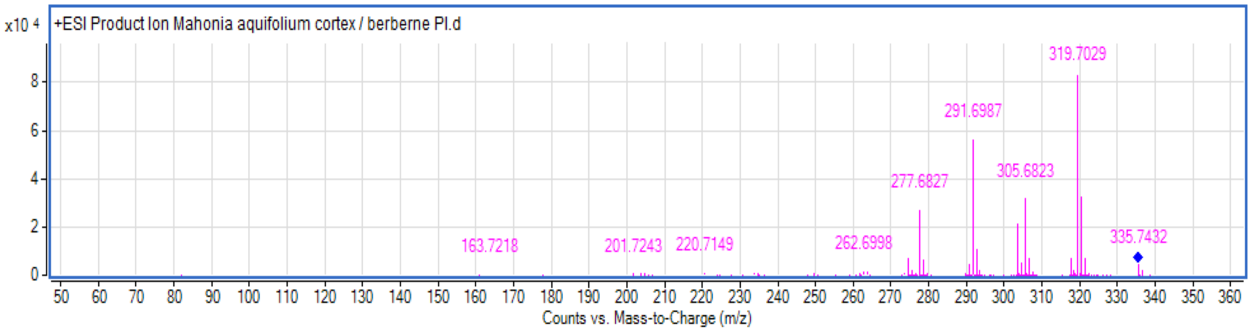

Figure S12B. MS spectrum obtained for berberine from *Mahonia aquifalium* cortex extract.

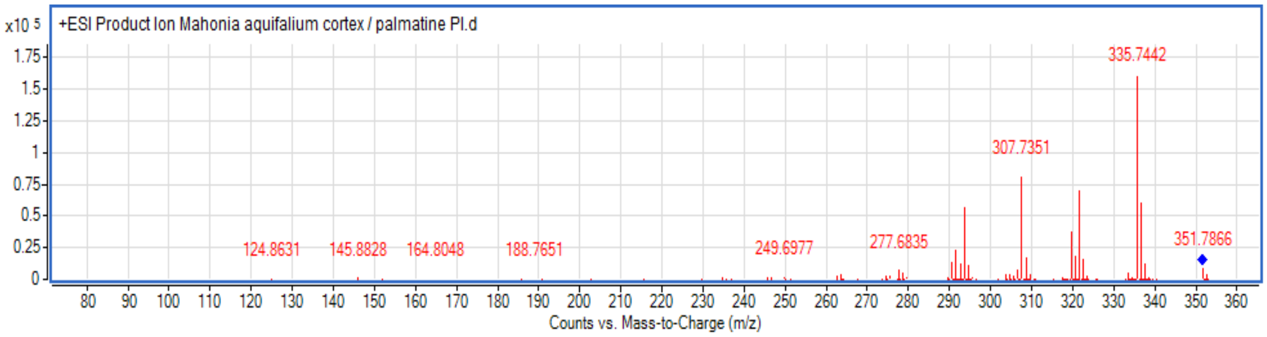

Figure S12C. MS spectrum obtained for palmatine from *Mahonia aquifalium* cortex extract.

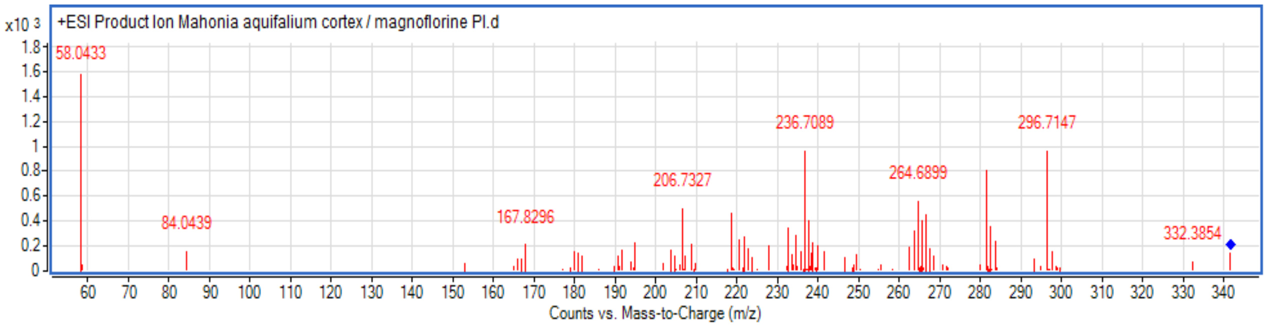

Figure S12D. MS spectrum obtained for magnoflorine from *Mahonia aquifalium* cortex extract.

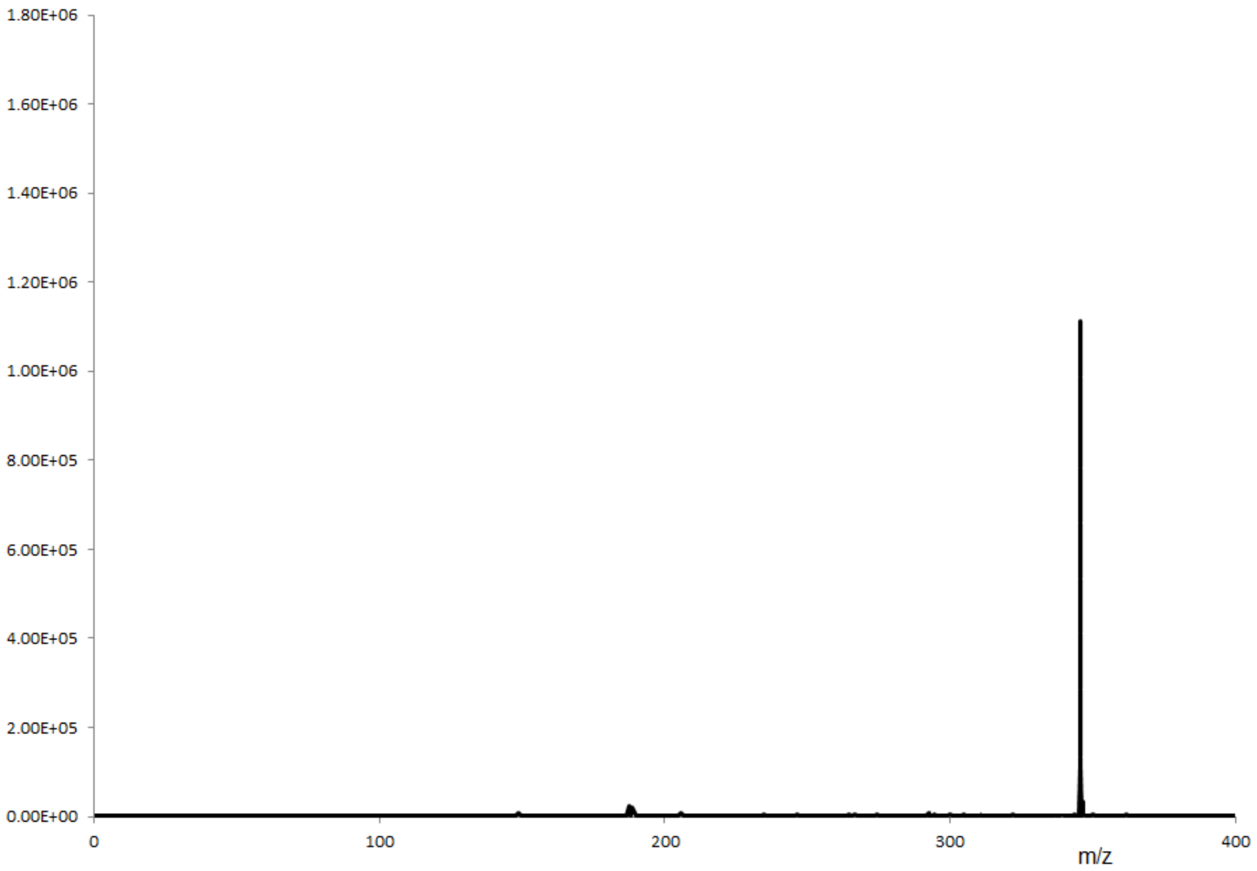

Figure S13A. MS spectrum obtained for *Mahonia aquifalium* leaves.

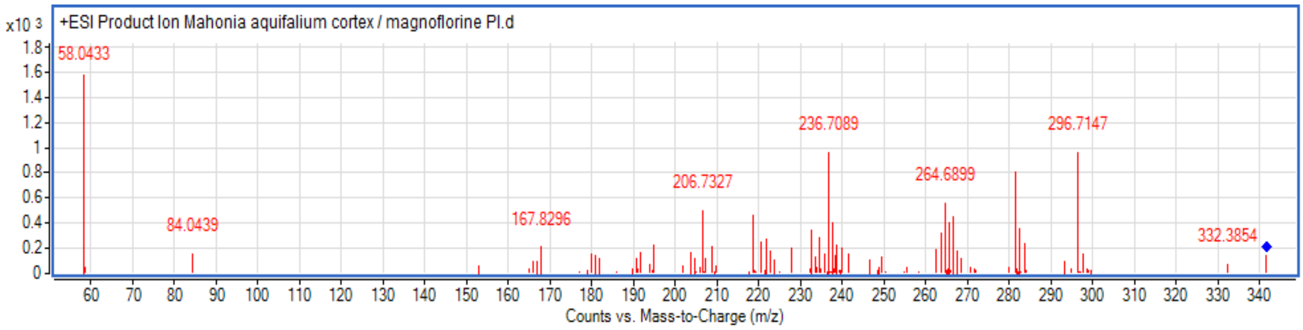

Figure S13B. MS spectrum obtained for magnoflorine from *Mahonia aquifalium* leaves.

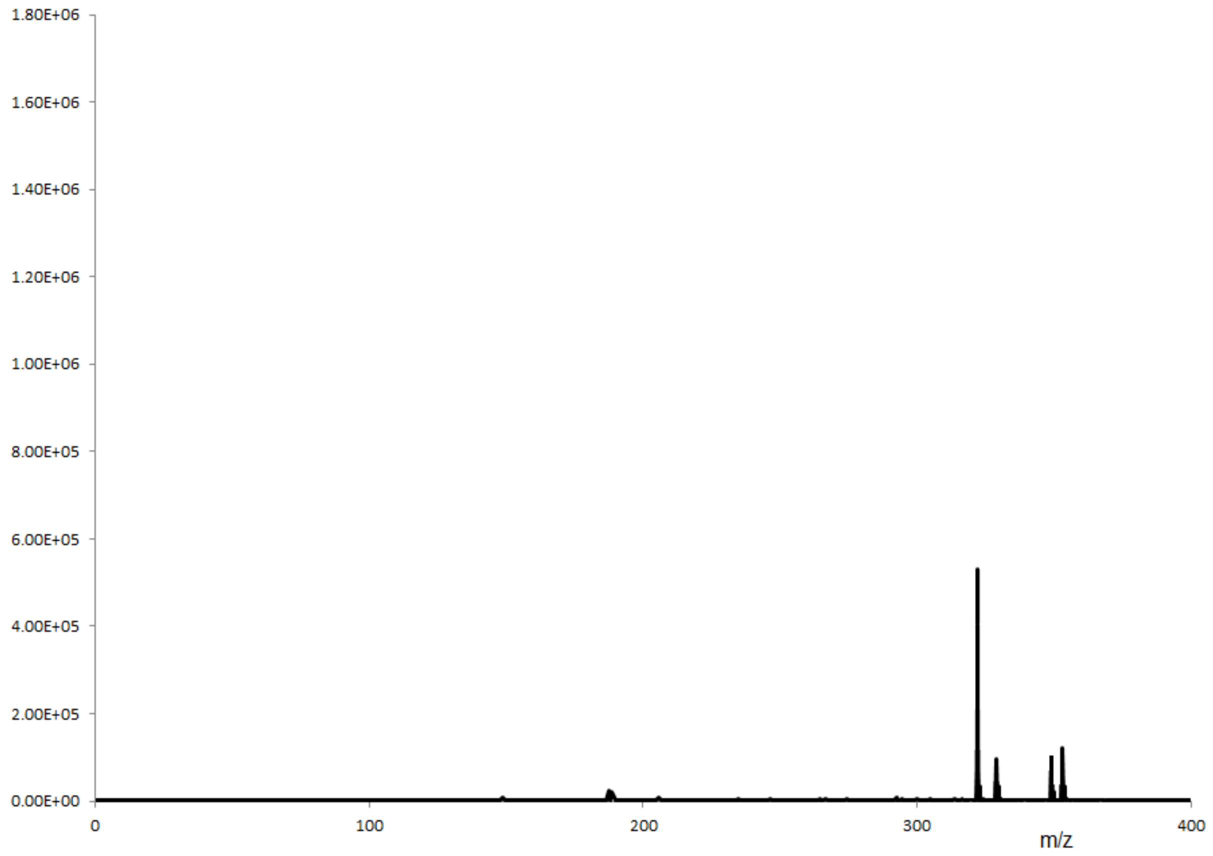

Figure S14A. MS spectrum obtained for *Fumaria officinalis* extract.

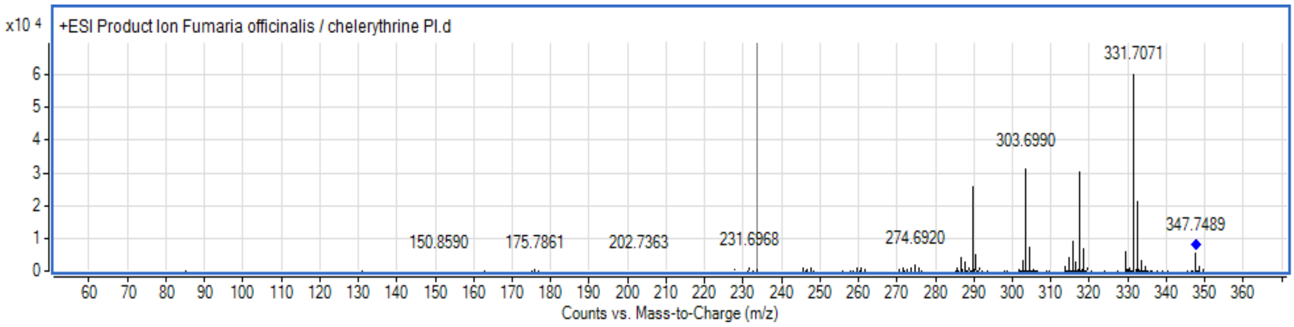

Figure S14B. MS spectrum obtained for chelerythrine from *Fumaria officinalis* extract.

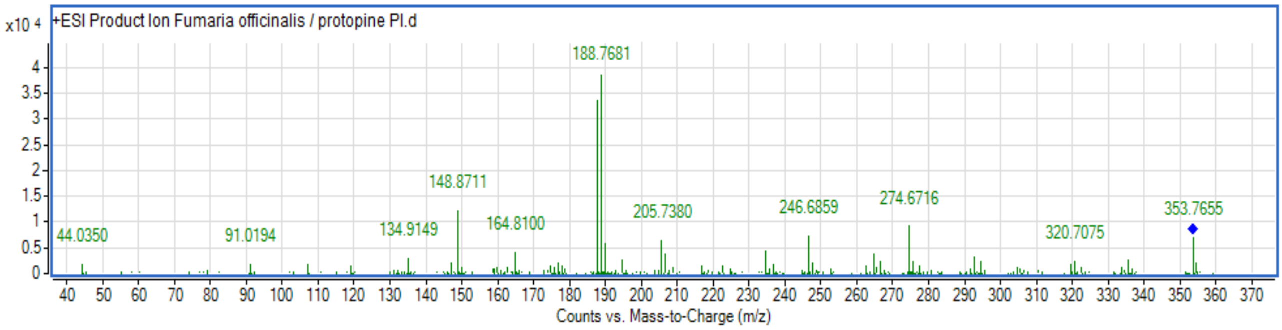

Figure S14C. MS spectrum obtained for protopine from *Fumaria officinalis* extract.

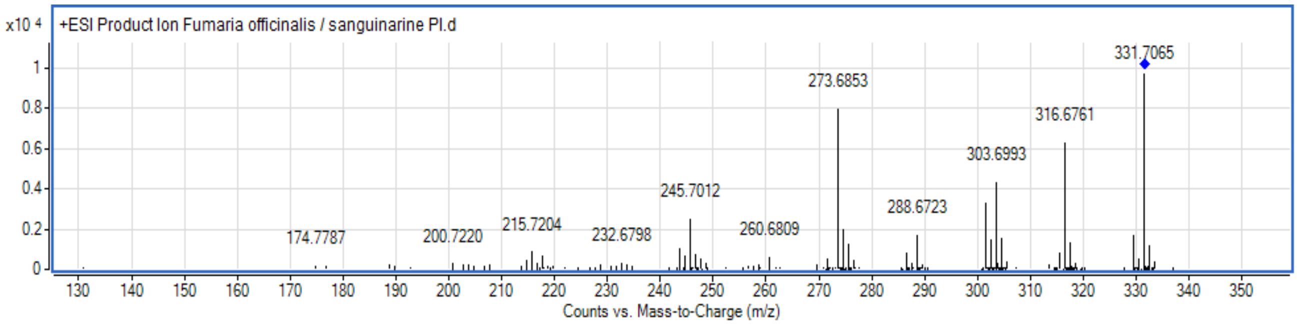

Figure S14D. MS spectrum obtained for sanguinarine from *Fumaria officinalis* extract.

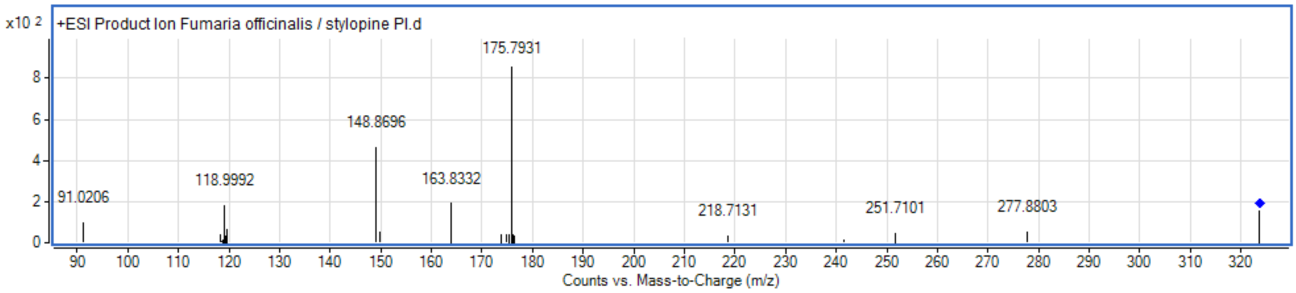

Figure S14E. MS spectrum obtained for stylopine from *Fumaria officinalis* extract.

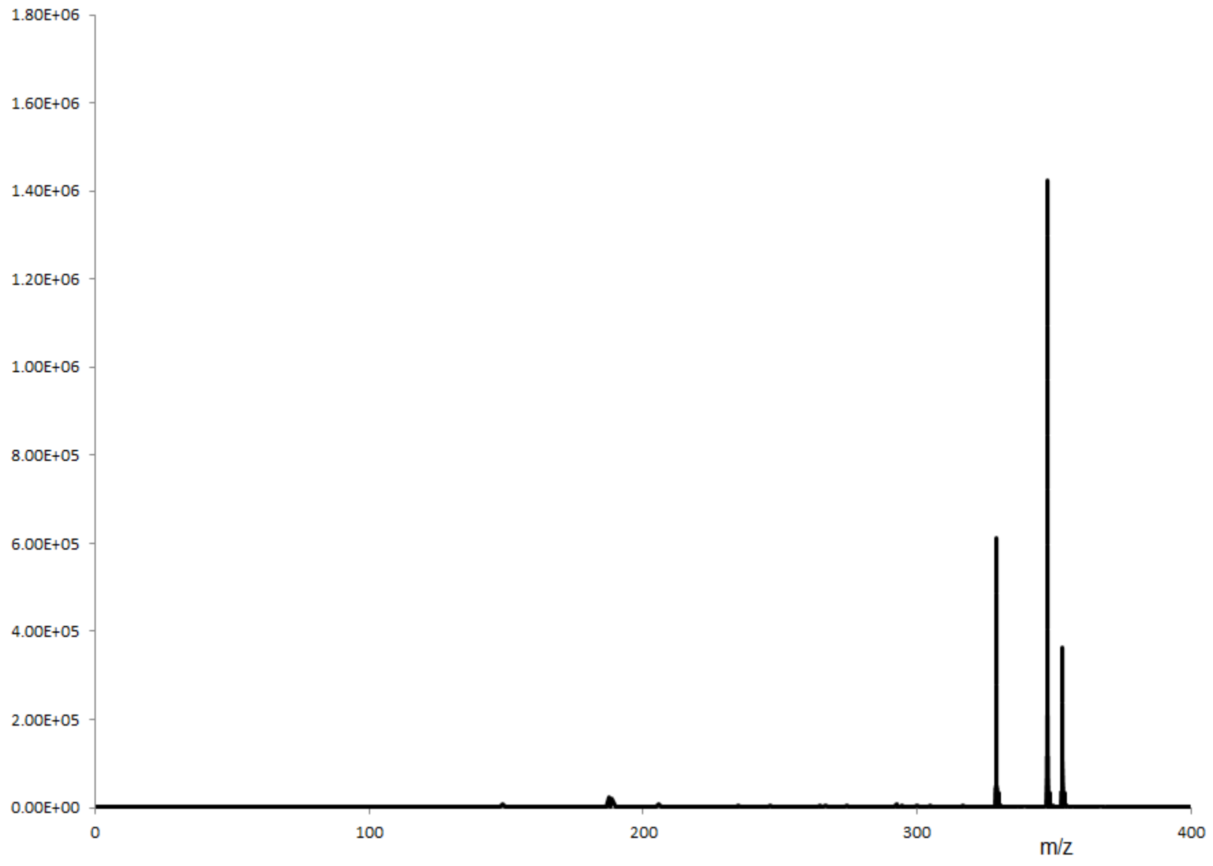

Figure S15A. MS spectrum obtained for *Macleaya cordata* leaves extract.

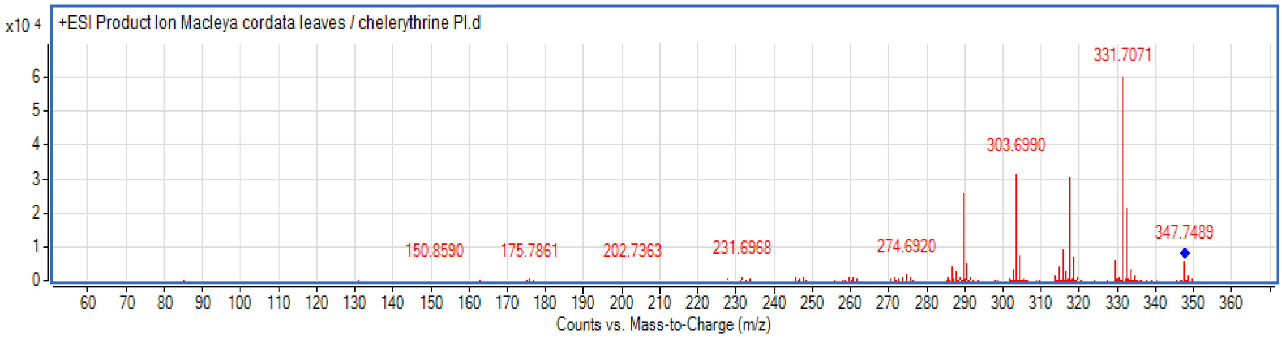

Figure S15B. MS spectrum obtained for chelerythrine from *Macleaya cordata* leaves extract.

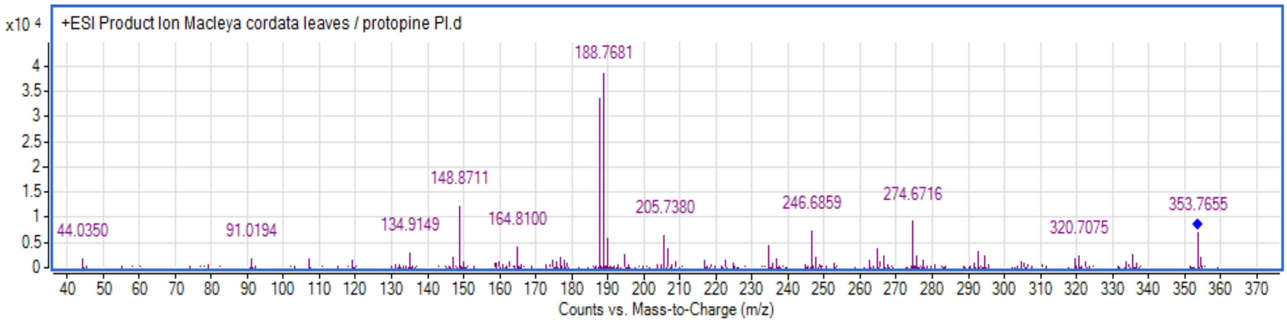

Figure S15C. MS spectrum obtained for protopine from *Macleaya cordata* leaves extract.

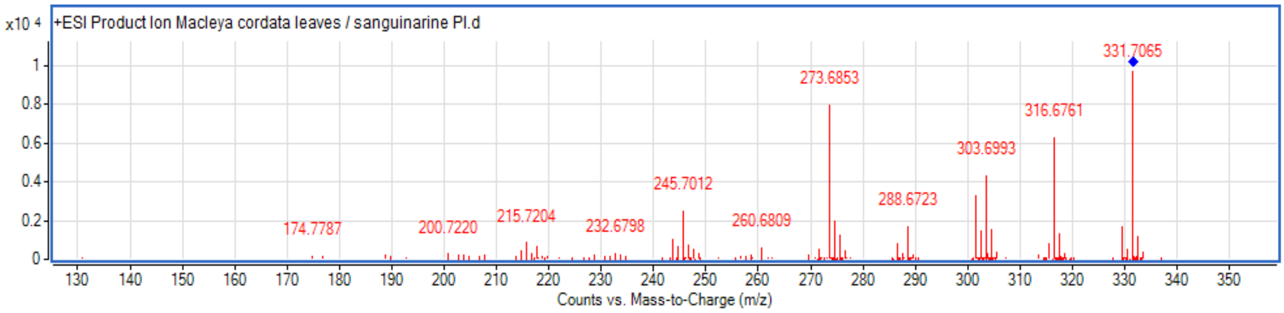

Figure S15D. MS spectrum obtained for sanguinarine from *Macleaya cordata* leaves extract.

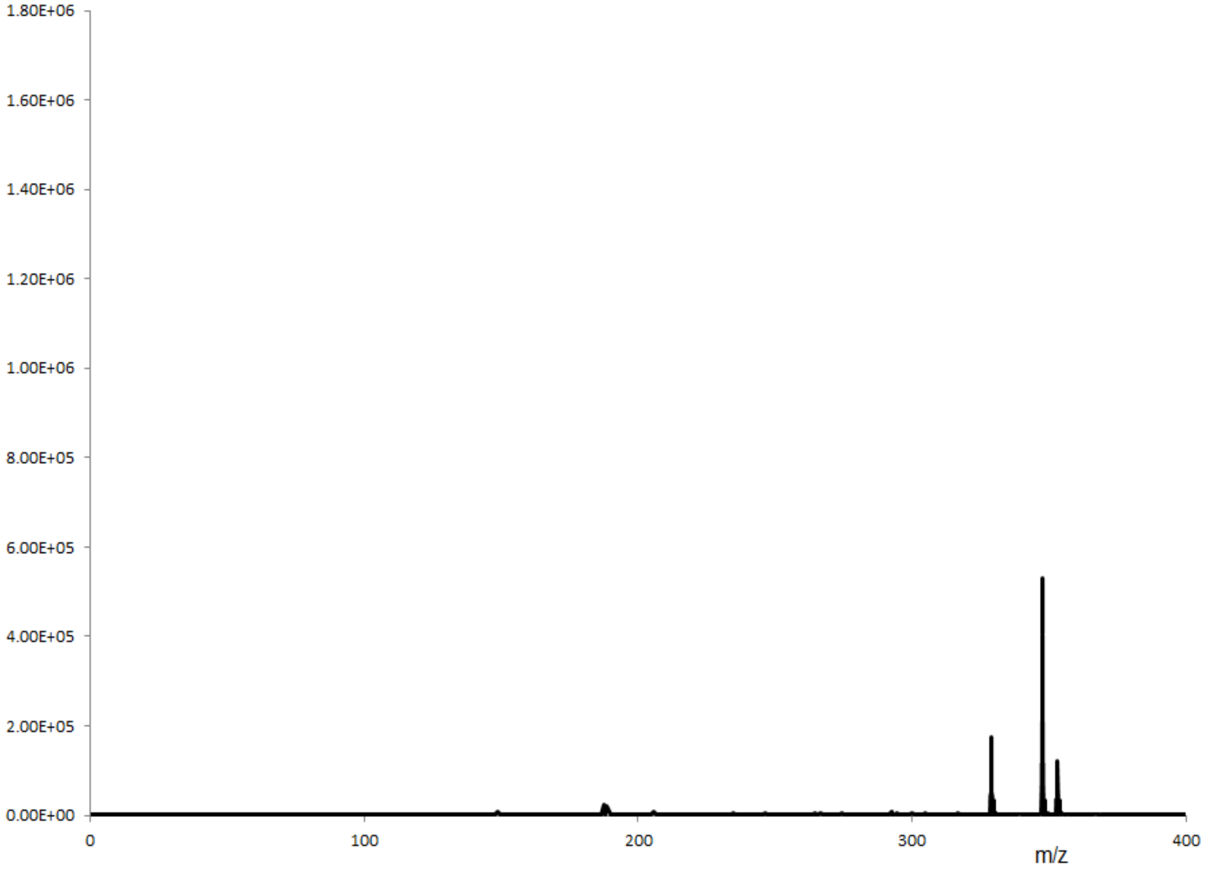

Figure S16A. MS spectrum obtained for chelerythrine from *Macleaya cordata* herb extract.

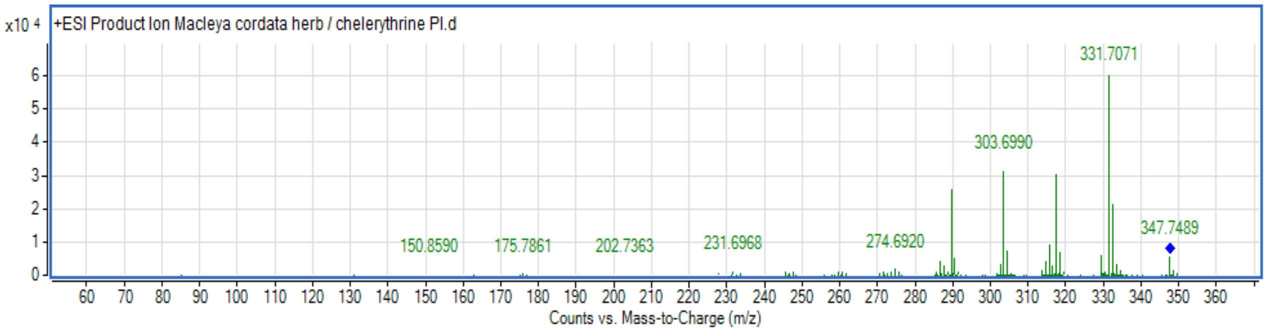

Figure S16B. MS spectrum obtained for chelerythrine from *Macleaya cordata* herb extract.

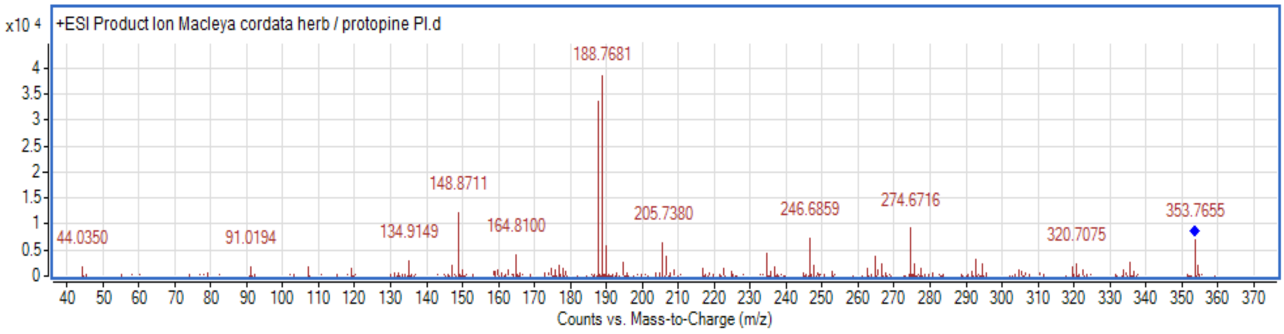

Figure S16C. MS spectrum obtained for protopine from *Macleaya cordata* herb extract.

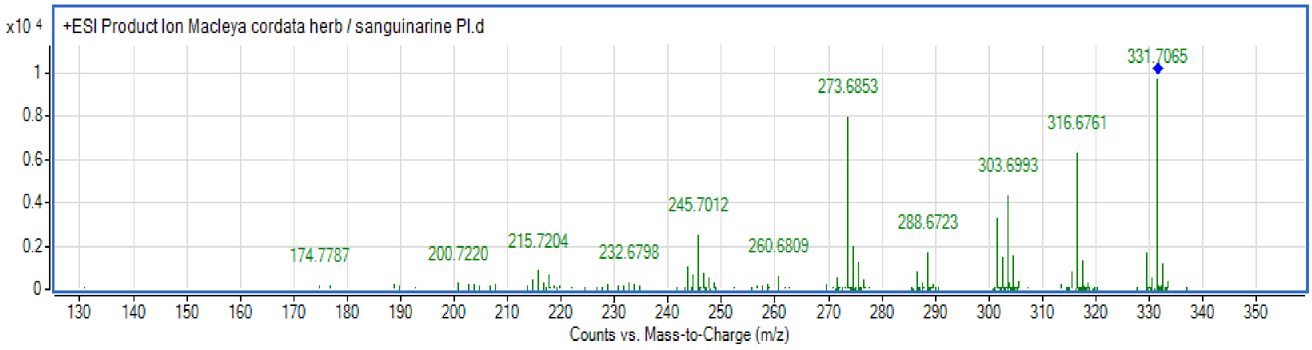

Figure S16D. MS spectrum obtained for sanguinarine from *Macleaya cordata* herb extract.

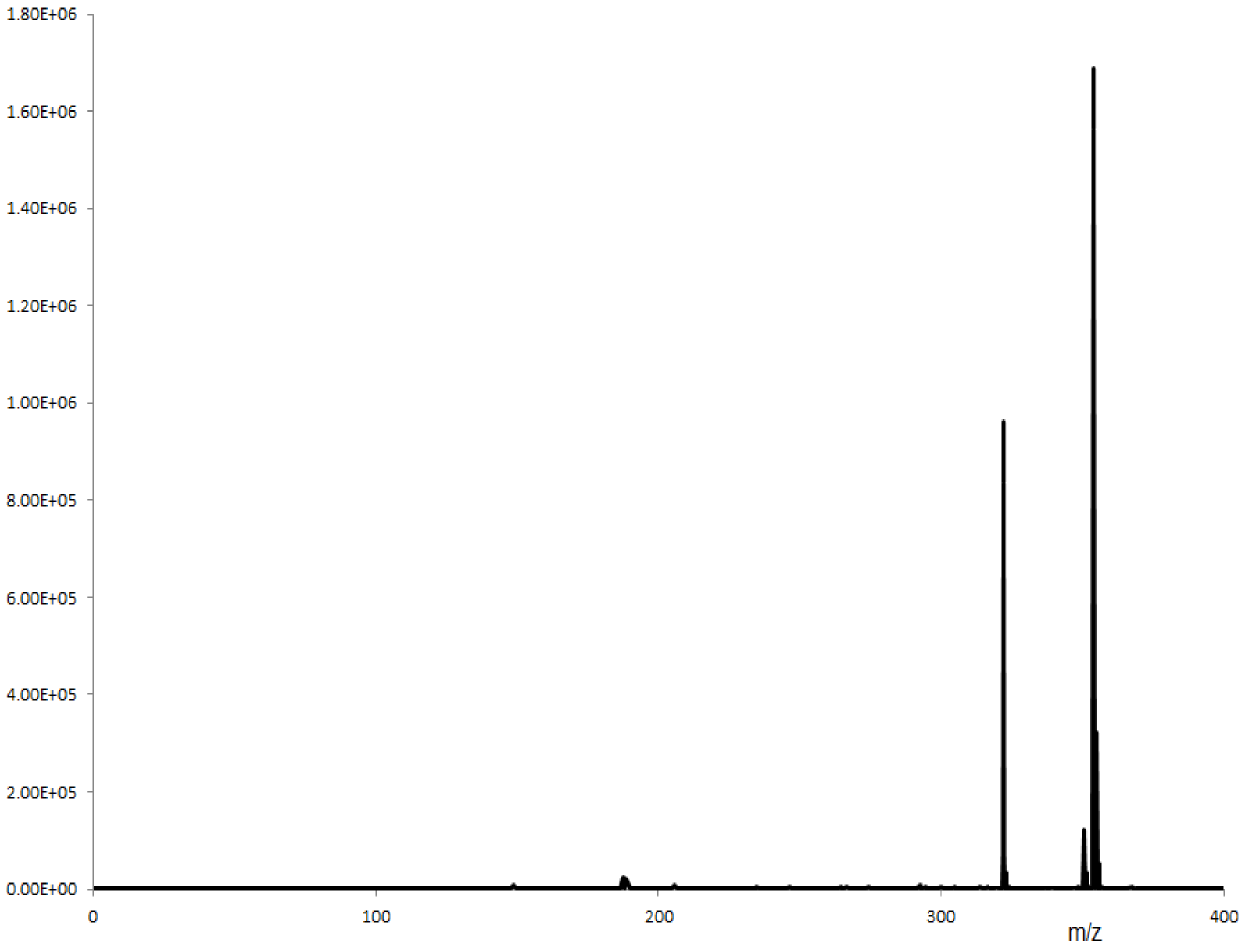

Figure S17A. MS spectrum obtained for *Corydalis lutea* root extract.

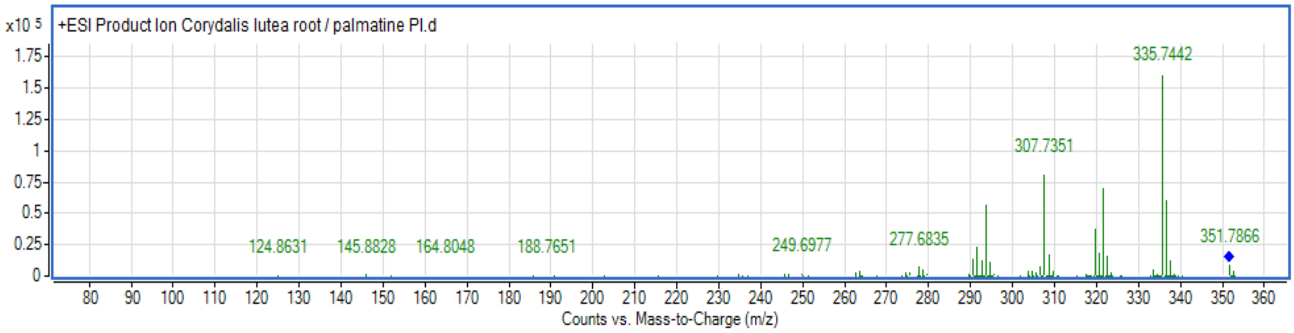

Figure S17B. MS spectrum obtained for palmatine from *Corydalis lutea* root extract.

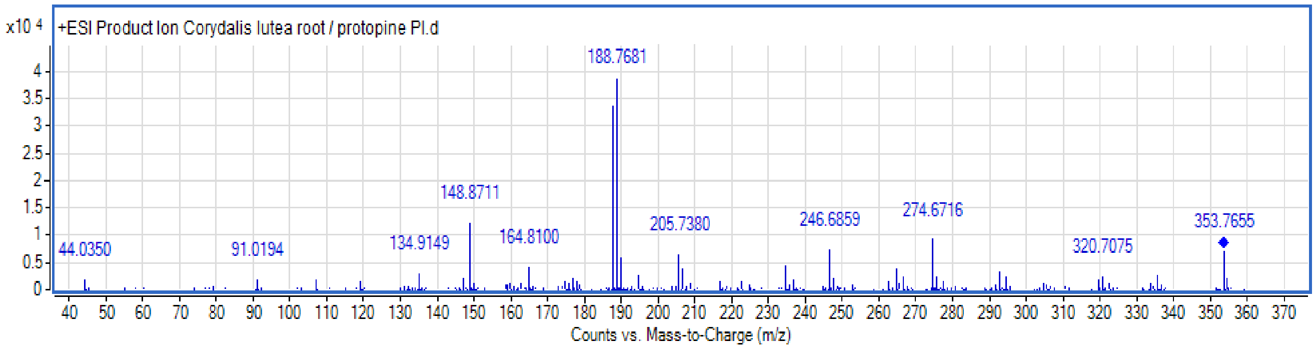

Figure S17C. MS spectrum obtained for protopine from *Corydalis lutea* root extract.

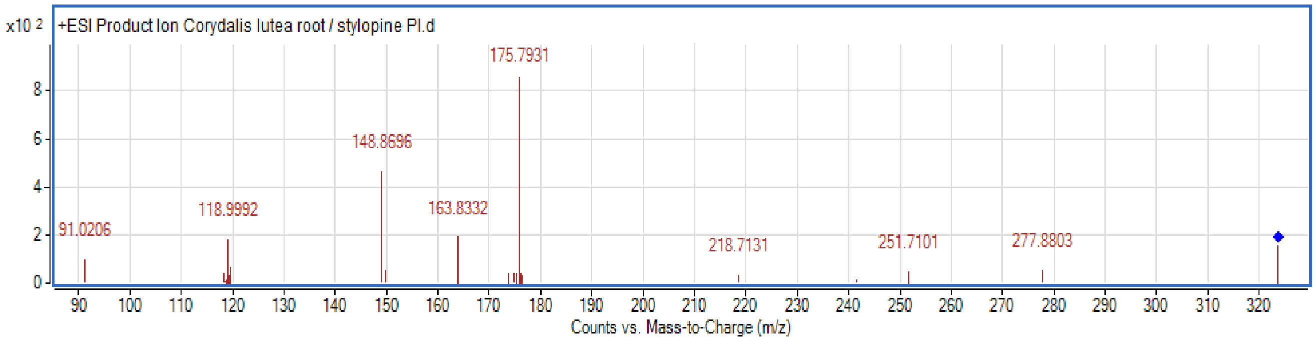

Figure S17D. MS spectrum obtained for stylopine from *Corydalis lutea* root extract.

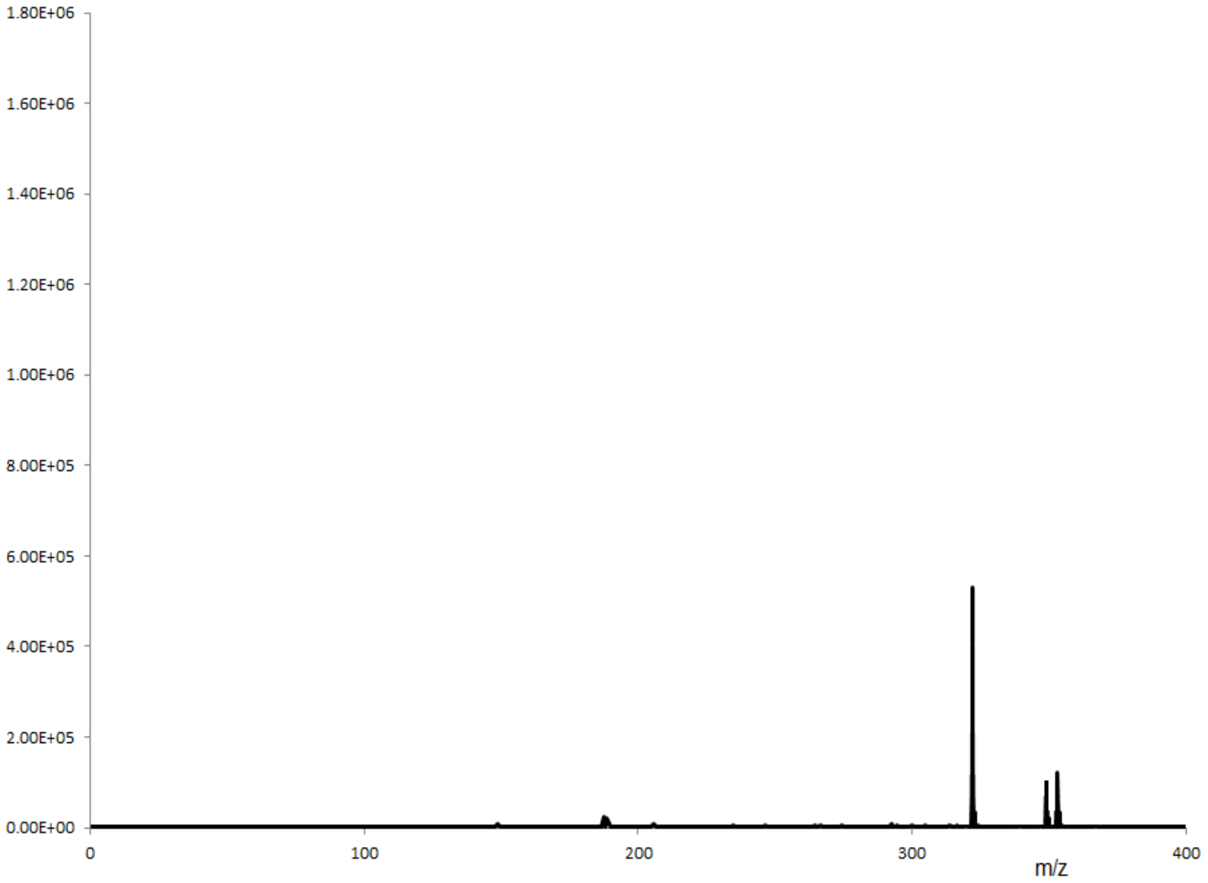

Figure S18A. MS spectrum obtained for *Corydalis lutea* herb extract.

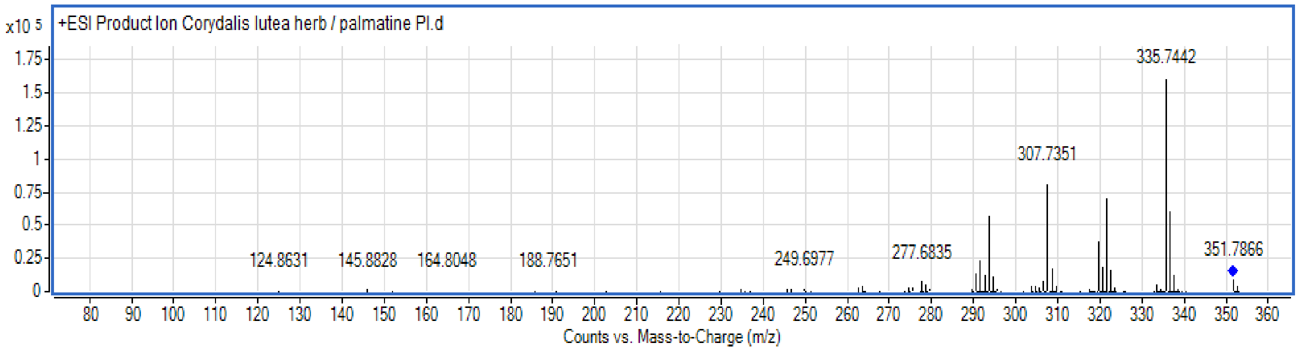

Figure S18B. MS spectrum obtained for palmatine from *Corydalis lutea* herb extract.

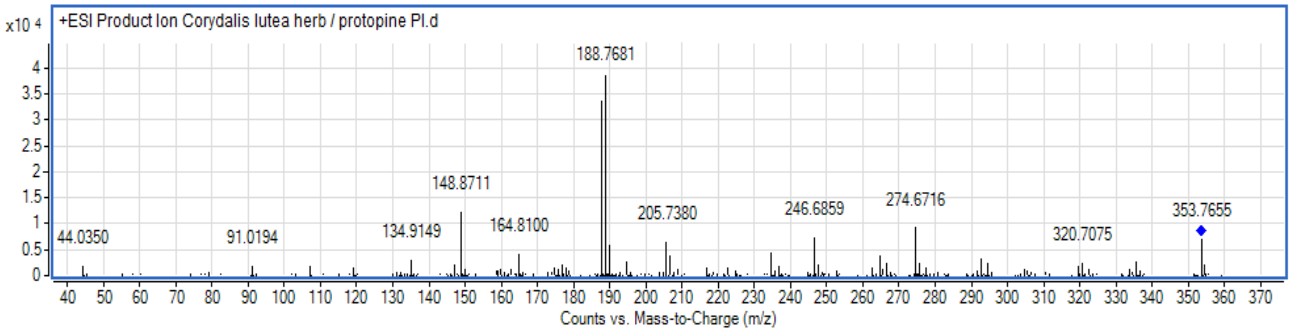

Figure S18C. MS spectrum obtained for protopine from *Corydalis lutea* herb extract.

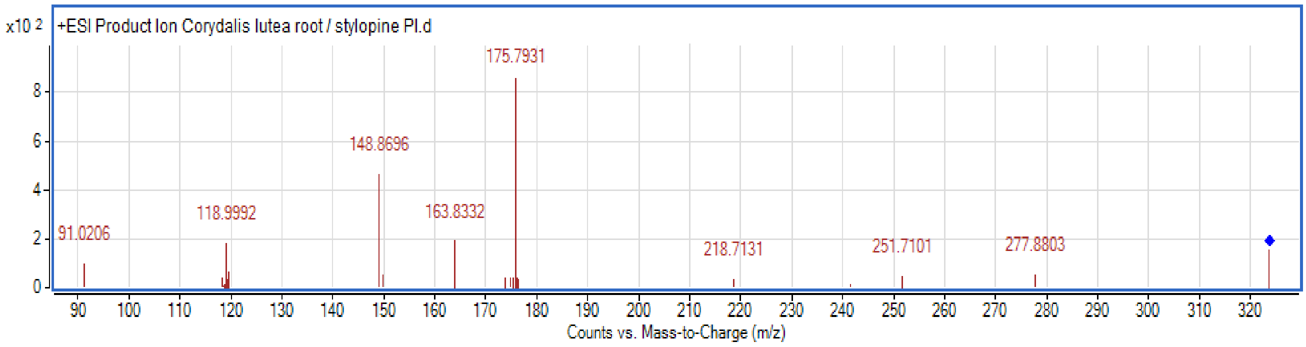

Figure S18D. MS spectrum obtained for stylopine from *Corydalis lutea* herb extract.

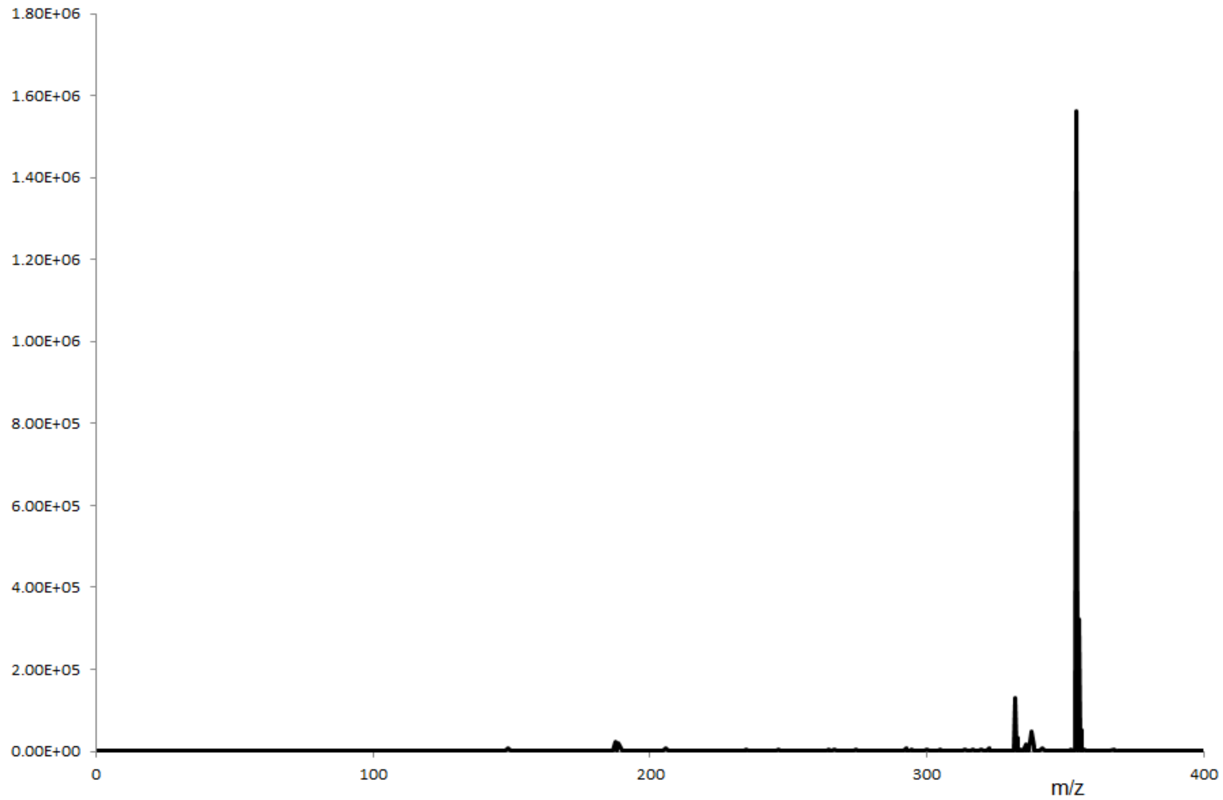

Figure S19A. MS spectrum obtained for *Dicentra spectabilis* herb extract.

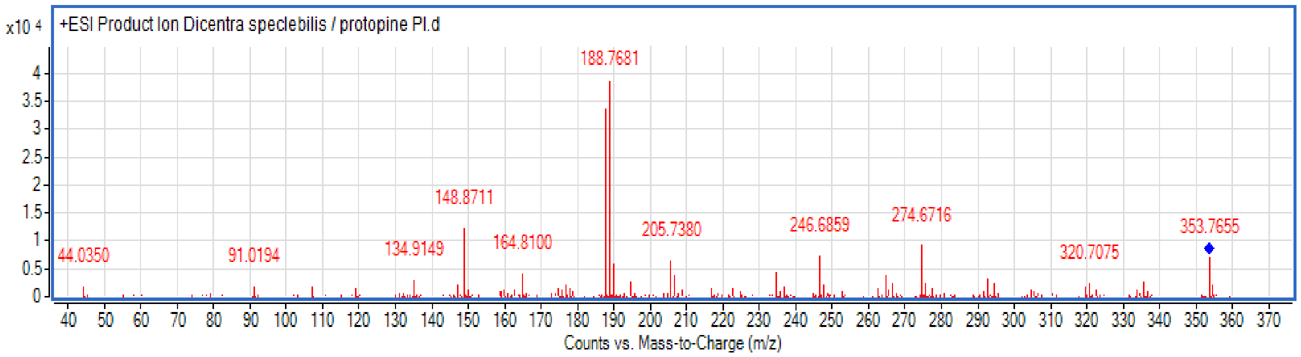

Figure S19B. MS spectrum obtained for protopine from *Dicentra spectabilis* herb extract.

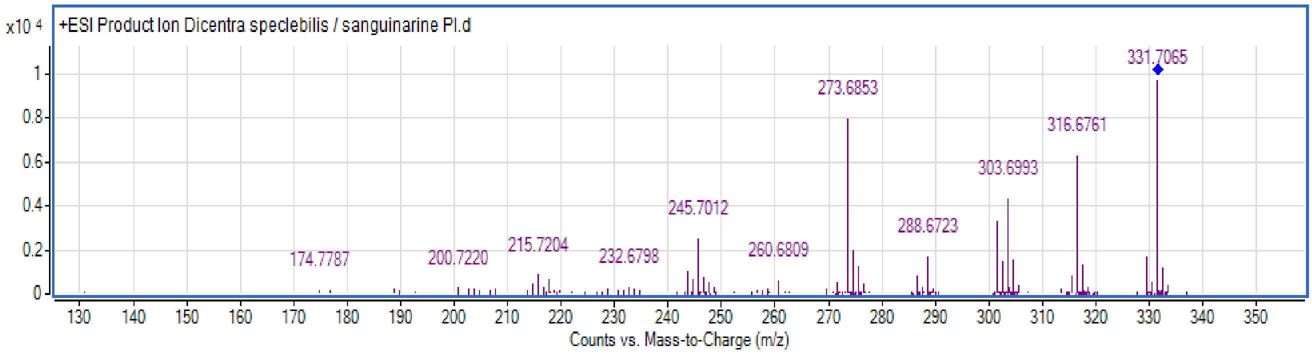

Figure S19C. MS spectrum obtained for sanguinarine from *Dicentra spectabilis* herb extract.

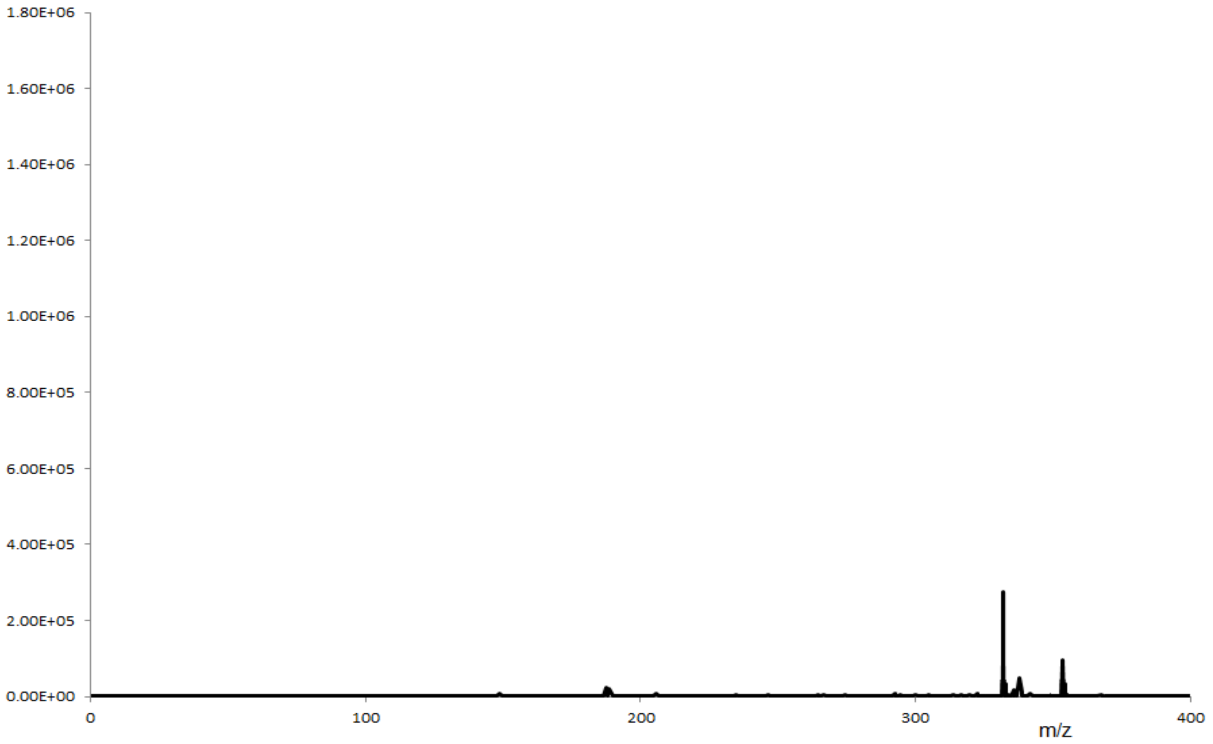

Figure S20A. MS spectrum obtained for *Meconopsis cambrica* root extract.

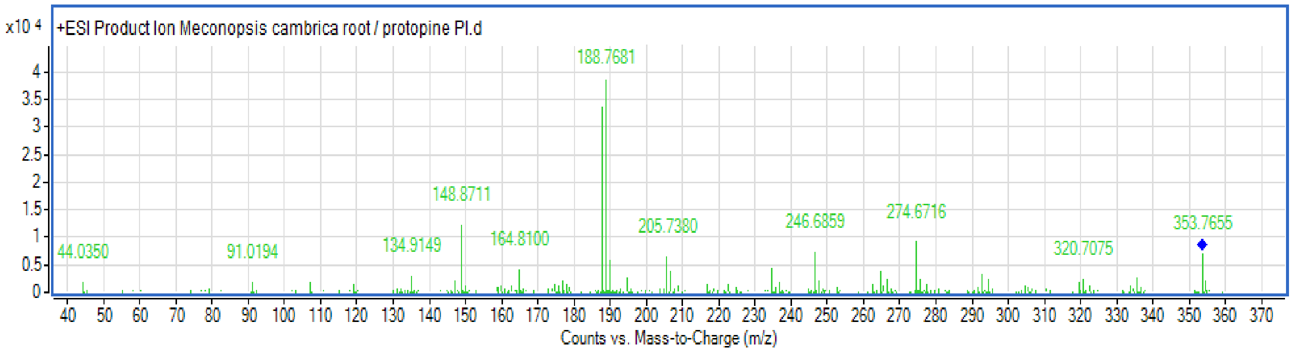

Figure S20A. MS spectrum obtained for protopine from *Meconopsis cambrica* root extract.

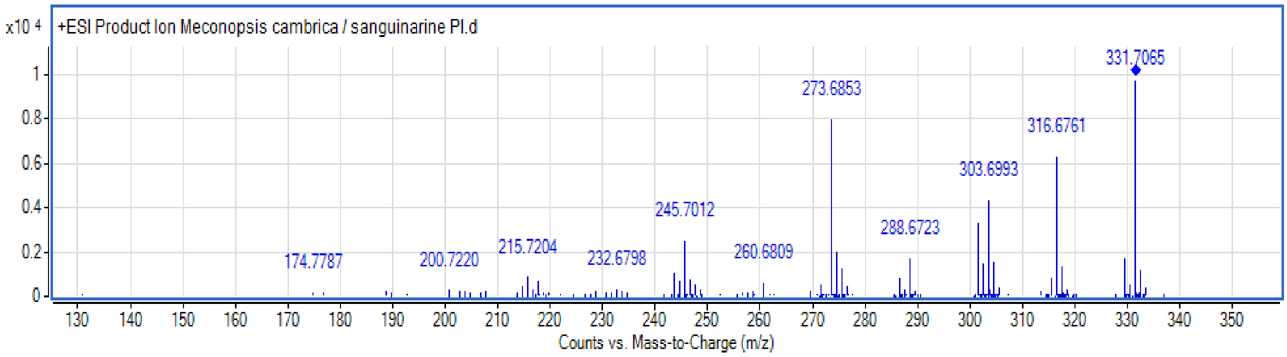

Figure S20B. MS spectrum obtained for sanguinarinee from *Meconopsis cambrica* root extract.

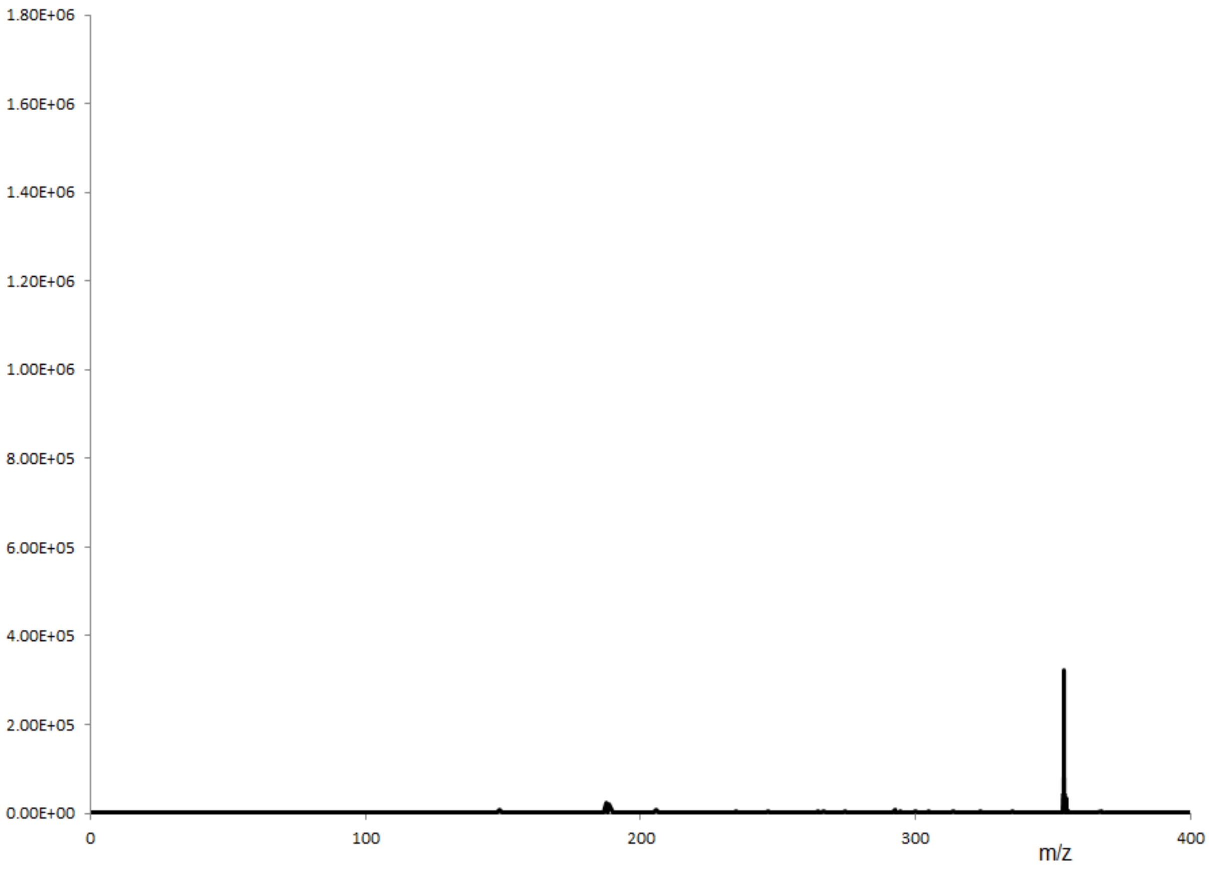

Figure S21A. MS spectrum obtained for *Meconopsis cambrica* herb extract.

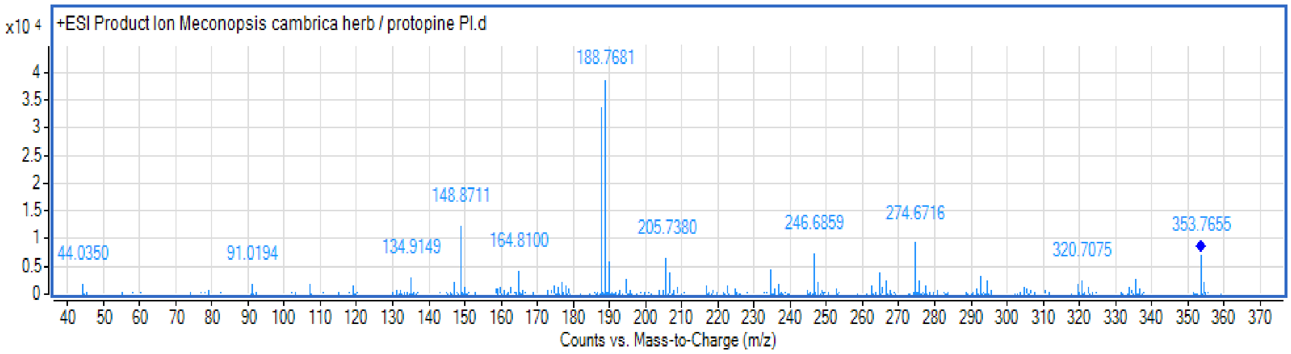

Figure S21A. MS spectrum obtained for protopine from *Meconopsis cambrica* herb extract.

Table S1. Exemplary of recoveries of extraction (%) obtained for alkaloids.

Plant extract	Berberine	Chelerythrine	Magnoflorine	Palmatine	Protopine	Sanguinarine	Stylopine
Corydalis lutea herb	87.4	97.2	90.4	86.9	101.2	98.5	90.7
Corydalis lutea root	92.1	94.2	89.5	90.9	102.1	96.5	94.7
Mahonia aquifalium cortex	92.4	93.1	88.6	83.2	95.4	104.6	92.1
